# Genomic analysis of a novel Neanderthal from Mezmaiskaya Cave provides insights into the genetic relationships of Middle Palaeolithic populations

**DOI:** 10.1038/s41598-022-16164-9

**Published:** 2022-07-29

**Authors:** Tatiana V. Andreeva, Andrey D. Manakhov, Fedor E. Gusev, Anton D. Patrikeev, Lyubov V. Golovanova, Vladimir B. Doronichev, Ivan G. Shirobokov, Evgeny I. Rogaev

**Affiliations:** 1 Center for Genetics and Life Science, Sirius University of Science and Technology, Sochi, Russia 354340; 2 Laboratory of Evolutionary Genomics, Department of Genomics and Human Genetics, Vavilov Institute of General Genetics, Russian Academy of Sciences, Moscow, Russia 119333; 3 Faculty of Biology, Centre for Genetics and Genetic Technologies, Lomonosov Moscow State University, Moscow, Russia 119192; 4 ANO Laboratory of Prehistory, St. Petersburg, Russia 190020; 5 Peter the Great Museum of Anthropology and Ethnography (Kunstkamera), Russian Academy of Sciences, St. Petersburg, Russia 199034; 6 Department of Psychiatry, University of Massachusetts Medical School, Worcester, MA 01604 USA

**Keywords:** Evolution, Genetics

## Abstract

The Mezmaiskaya cave is located on the North Caucasus near the border that divides Europe and Asia. Previously, fossil remains for two Neanderthals were reported from Mezmaiskaya Cave. A tooth from the third archaic hominin specimen (*Mezmaiskaya 3*) was retrieved from layer 3 in Mezmaiskaya Cave. We performed genome sequencing of *Mezmaiskaya 3.* Analysis of partial nuclear genome sequence revealed that it belongs to a *Homo sapiens neanderthalensis* female*.* Based on a high-coverage mitochondrial genome sequence, we demonstrated that the relationships of *Mezmaiskaya 3* to *Mezmaiskaya 1* and *Stajnia S5000* individuals were closer than those to other Neanderthals. Our data demonstrate the close genetic connections between the early Middle Palaeolithic Neanderthals that were replaced by genetically distant later group in the same geographic areas. Based on mitochondrial DNA (mtDNA) data, we suggest that *Mezmaiskaya 3* was the latest Neanderthal individual from the early Neanderthal’s branches. We proposed a hierarchical nomenclature for the mtDNA haplogroups of Neanderthals. In addition, we retrieved ancestral mtDNA mutations in presumably functional sites fixed in the Neanderthal clades, and also provided the first data showing mtDNA heteroplasmy in Neanderthal specimen.

## Introduction

Since the late Marine Isotope Stage (MIS) 9, Neanderthals occupied regions in West Eurasia that were most affected by climatic fluctuations during the glacial and interglacial cycles of MIS 8–MIS 3. In Central Europe and Eastern Europe, the Micoquian stone-working bifacial tradition^1^ lasted from at least MIS 5 to the end of Middle Palaeolithic (MP) in MIS 3, and was widespread in Central and Eastern Europe^2–4^. Palaeogenetic research of Neanderthal mitochondrial DNA (mtDNA) identified at least two population turnovers that occurred during Neanderthal history and both were associated with the Micoquian. The earlier population turnover at ~ 90 ka was related to the replacement of the earlier Altai Neanderthals by western European Neanderthals, which was associated with the spread of the Eastern Micoquian to the Altai region^5,6^. The later population turnover occurred towards the end of Neanderthal history from 47 to 39 ka within the Eastern Micoquian Neanderthal population in the North Caucasus or among late Middle Palaeolithic (LMP) Neanderthal groups throughout Europe^7^. Palaeogenetic research also detected a great similarity between the mitochondrial genomes of two Neanderthals from geographically distant Eastern Micoquian contexts dated to MIS 5—Stajnia Cave (Poland) and Mezmaiskaya Cave (Russia)—and both of them fall outside of the mtDNA variation of the later European Neanderthals^4^.

**Figure 1:**
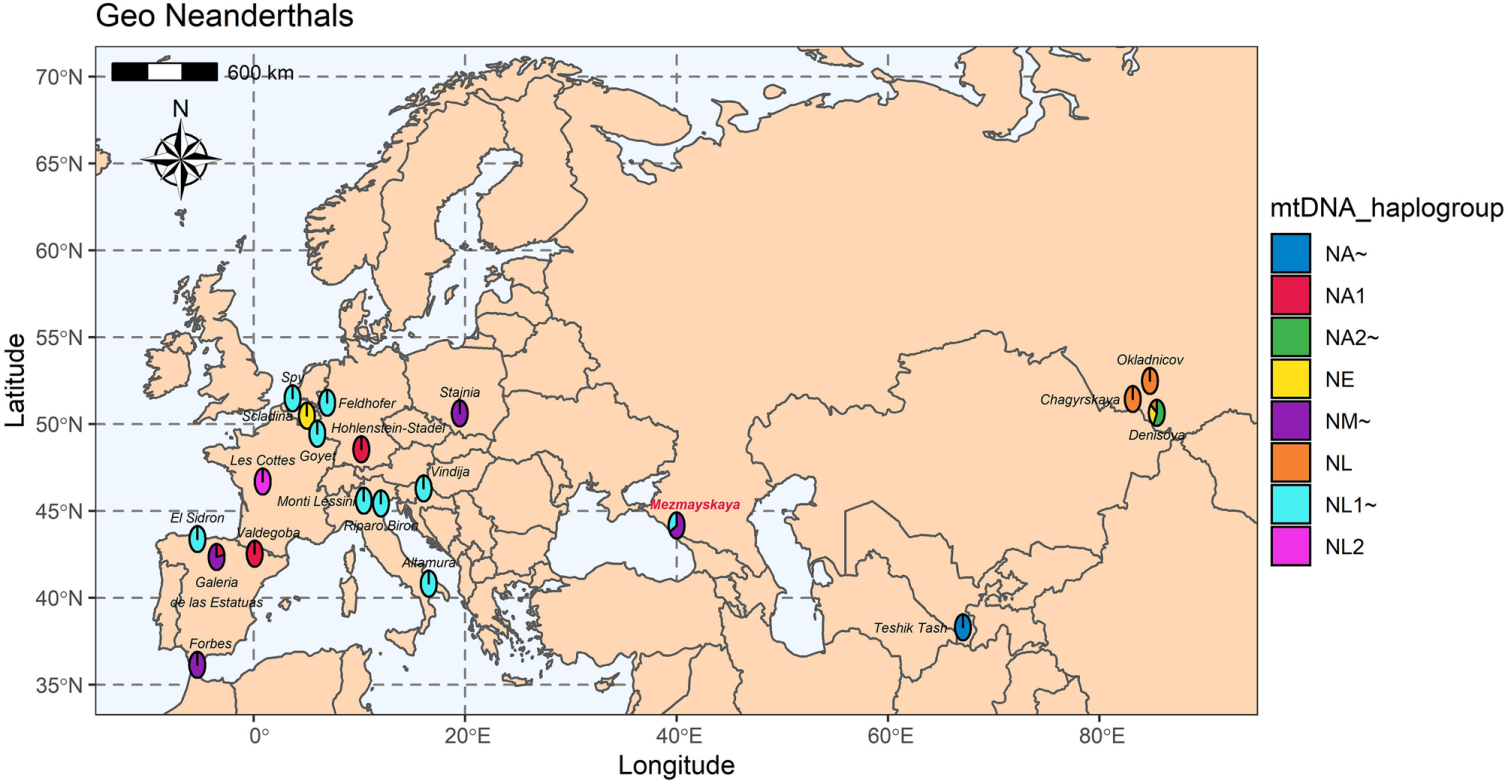
Eurasia map showing the locations of the Neanderthal samples used in the analysis. Mezmaiskaya Cave is typed in red. Neanderthal haplogroups (see *Results,* Supplementary Fig. S10) are indicated at each location by coloured sectors, and large haplogroups combine subsequent branches: NA2 ~ includes NA2 and NA2a; NM1 ~ includes NM1, NM1a, NM2; NL1 ~ includes NL1a1, NL1a2a, NL1a2b1, NL1a2b. NA ~ *Teshik-Tash* individual. The figure was generated using ggplot2 (https://ggplot2.tidyverse.org), ggspatial (https://github.com/paleolimbot/ggspatial), rnaturalearth (https://github.com/ropensci/rnaturalearth), rnaturalearthdata (https://github.com/ropensci/rnaturalearthdata), scatterpie (https://CRAN.R-project.org/package=scatterpie) and sf (https://github.com/r-spatial/sf) packages for R.

The Mezmaiskaya Cave, located in Russia, is widely known as a Palaeolithic site that has recorded the longest MP and Upper Palaeolithic sequences in the North Caucasus area (Fig. 1, see SI, Supplementary Figs. S1–S3). In the MP deposits, two Neanderthal fossils were found in this cave, namely, a newborn skeleton (*Mezmaiskaya 1*) that was found in the lowermost Eastern Micoquian level (layer 3) and a juvenile (*Mezmaiskaya 2*) that was found in the uppermost Eastern Micoquian level (layer 2)^4,7–13^. Here, we present mtDNA and genome sequencing results on a new Eastern Micoquian Neanderthal individual (*Mezmaiskaya 3*) based on a milk incisor found in layer 3 at the Mezmaiskaya Cave.

## Results

### Anthropological definition of Mezmayskaya 3

**Figure 2:**
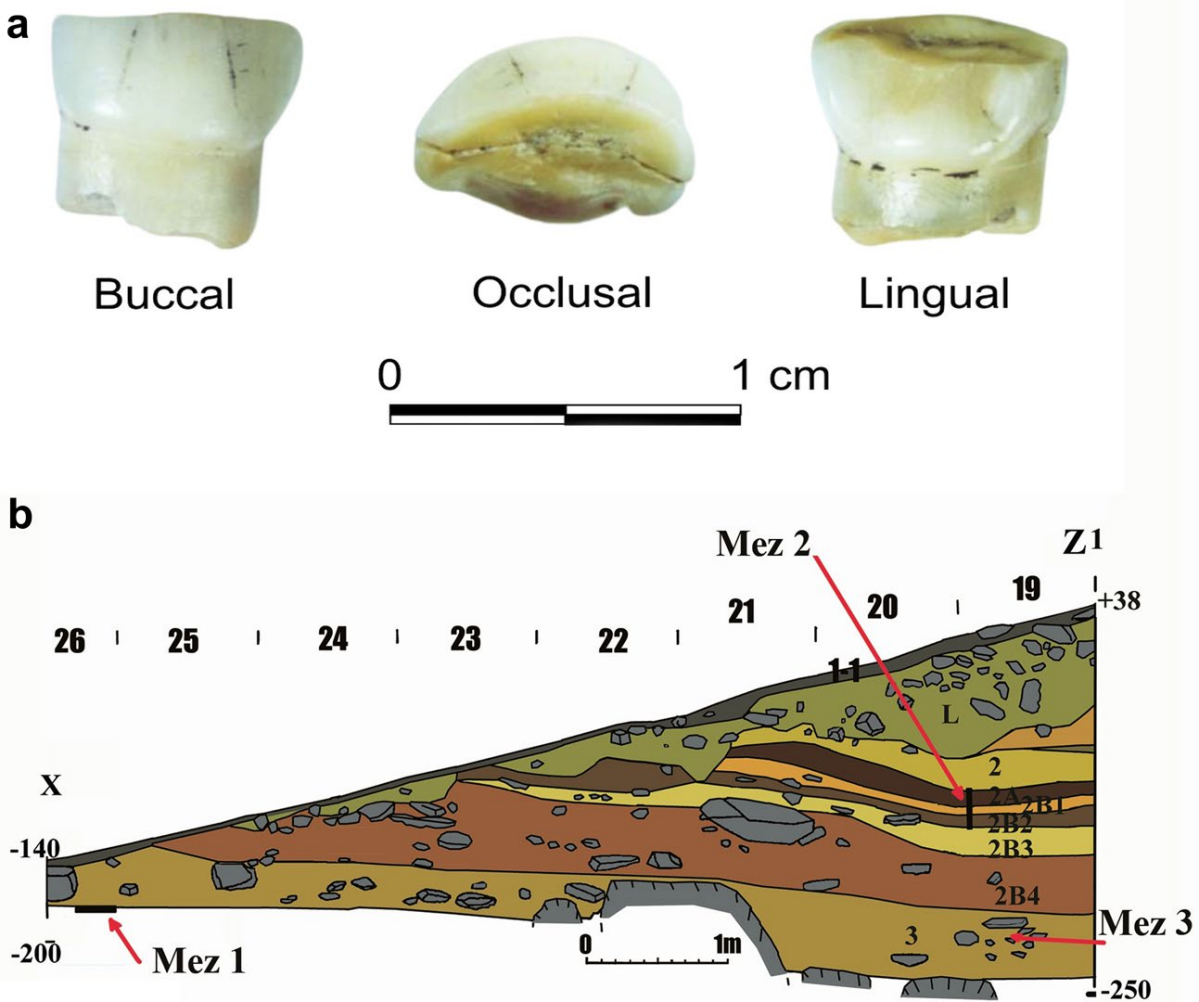
(**a**) Neanderthal incisor (*Mezmaiskaya 3*) from Mezmaiskaya Cave. (**b**) General longitudinal profile XZ1 indicating the stratigraphic position of anthropological finds: *Mezmaiskaya 1* (Mez1), *Mezmaiskaya 2* (Mez2)*,* and *Mezmaiskaya 3* (Mez3).

*Mezmaiskaya 3* tooth morphology suggested that it may belong to archaic hominins (Fig. 2). While the tooth crown is strongly worn, morphological features suggest that this is a milk right, upper central incisor (Rdi^1^). The crown of *Mezmaiskaya 3* is completely formed, and the wear of the crown approximately corresponds to the wear of the central incisor No. 32 of the child from Renne Grotto at Arcy-sur-Cure, whose age was estimated at 5–6 years old^14^. Based on this comparison, we assumed that the *Mezmaiskaya 3* individual was about the same age.

### Data generation and basic processing

DNA was extracted from the *Mezmaiskaya 3* (*Mez3*) tooth (Supplementary Fig. S4) and converted into a single-stranded library for sequencing^15^. Almost half a billion raw DNA sequences were generated (Supplementary Table S1) by sequencing on the Illumina HiSeq 2000/2500. DNA fragments were mapped to the human reference genome (GRCh37) as well as to the revised Cambridge Reference Sequence (rCRS, NC_012920.1) of the human mitochondrial genome and the Neanderthal mitochondrial genome sequence (NC_011137.1) (see *Materials and Methods* for details, Supplementary Tables S2, S5). C-to-T base substitutions at the ends of the DNA sequences in the mapped sequences were related to the postmortem degradation pattern of ancient DNA (Supplementary Fig. S5). A total of 75 Mbp of nuclear genomic sequences with a signature of deamination mapped to the human genome were recovered (Supplementary Table S2). Based on the coverage of the X chromosome and autosomes, the *Mezmaiskaya 3* specimen was inferred to be female (Supplementary Table S3).

### mtDNA genome reconstruction

Mitochondrial DNA contamination was estimated at 2% (95% CI 1–3%). We reconstructed the complete mtDNA sequence from a consensus call, and most sequences at each mtDNA position carried the same allele, except a single mitochondrial position 8839 of the human reference genome with both A and G variants found in *Mezmaiskaya 3* individuals (Figure S8A, Supplementary Table S7). The 8839 A/G occurred at a ratio of ~ 1:2 and was replicated in the independent libraries. The 8839 A/G leads to the p.105Ala/Thr variant in the mitochondrial ATP synthase subunit 6 gene, which apparently represents mtDNA heteroplasmy in the *Mezmaiskaya 3* Neanderthal specimen. We also identified four variants specific for *Mezmaiskaya 3* mtDNA that were not presented in other Neanderthals. These variants have occurred in modern humans with low frequency (less than 0.02%) and are absent in all other archaic humans (Supplementary Fig. S8, Supplementary Table S6).

### Relationship of Mezmaiskaya 3 to other Neanderthals and age estimation

We performed phylogenetic analyses using the coding region of the mitochondrial genome for *Mezmaiskaya 3* and 27 previously published Neanderthals, 4 Denisovans, *Homo sapiens heidelbergensis*^16^, 10 ancient and 53 modern humans, and chimpanzees (see *Materials and Methods*). The maximum parsimony tree (Supplementary Fig. S9) of these sequences indicated that *Mezmaiskaya 3* has a Neanderthal-like mitochondrial genome and is closest to *Mezmaiskaya 1*^13^ from the same archaeological site. These two mitochondrial genomes and the *Stajnia S5000* individual from Poland belong to a mtDNA clade segregated from the *Altai*, *Denisova15,* and *Scladina I-4A* mitotypes.

**Figure 3:**
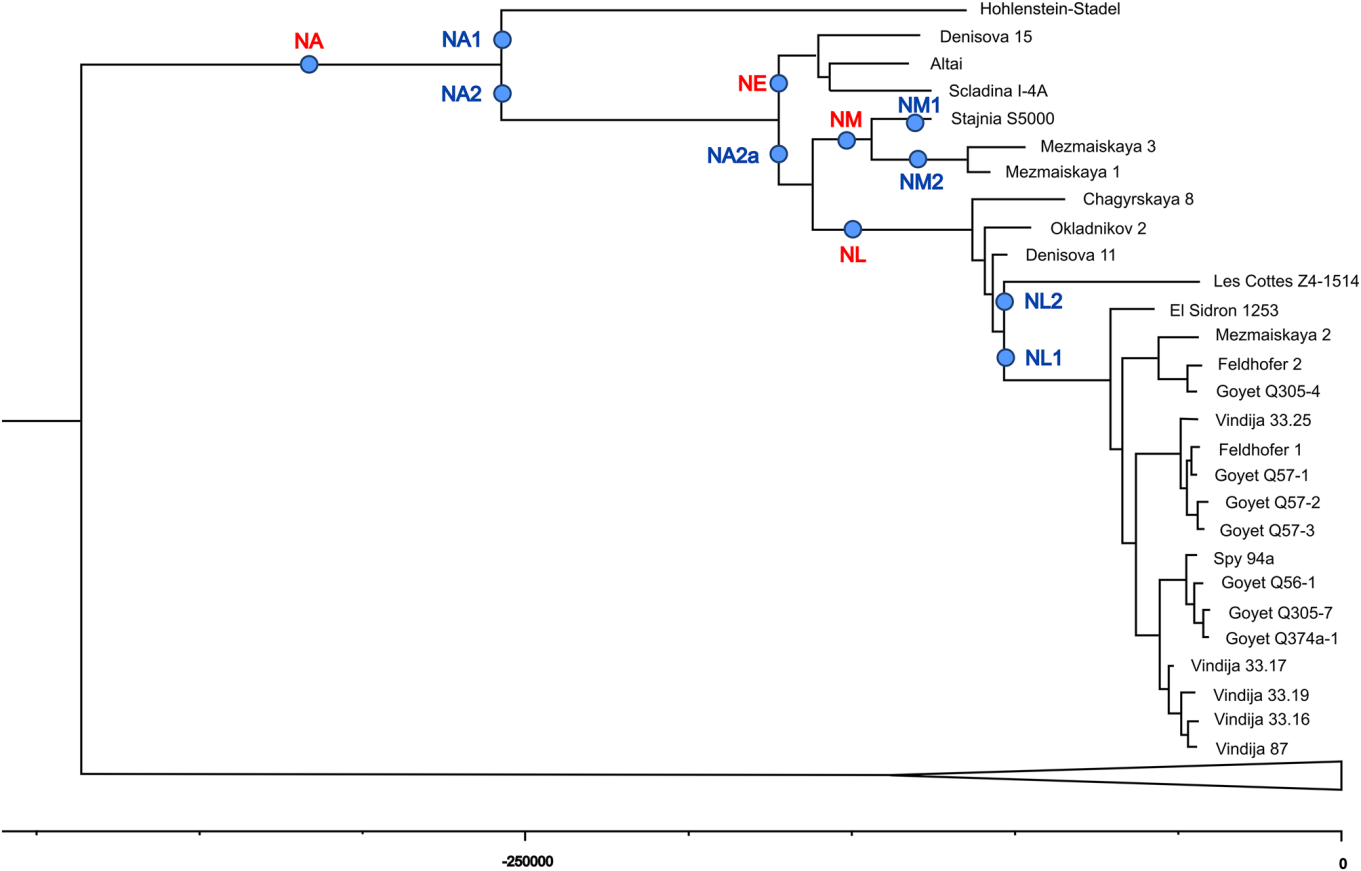
Bayesian phylogenetic tree relating the *Mezmaiskaya 3* to other archaic and modern human mitochondrial genomes. The branches corresponding to modern human mtDNAs are collapsed at the bottom of the figure. The tree was reconstructed by only using coding sequences. Neanderthal's haplogroups are indicated for the main branches.

We also performed Bayesian tree inference in a subset of ancient and modern genomes, which includes coding region data from directly radiocarbon-dated or present-day human samples (*Materials and Methods*, Fig. 3). We observed the same phylogenetic placement of the *Mezmaiskaya 3* genome as in the maximum parsimony tree. We also estimated the age of the *Mezmaiskaya 3* specimen to be 96.7 Kya (95% HPD interval from 59 to 134 kya) (Supplementary Table S12), which placed it within the 95% confidence intervals of both the *Mezmaiskaya 1* and *Stajnia S5000* mitochondrial genomes. The mtDNA sequences of these early Micoquian Neanderthals that lived ~ 100–70 kya ago are genetically distant from the later Micoquian European Neanderthals that lived ~ 60–40 kya ago.

### Nuclear genome analysis

**Figure 4:**
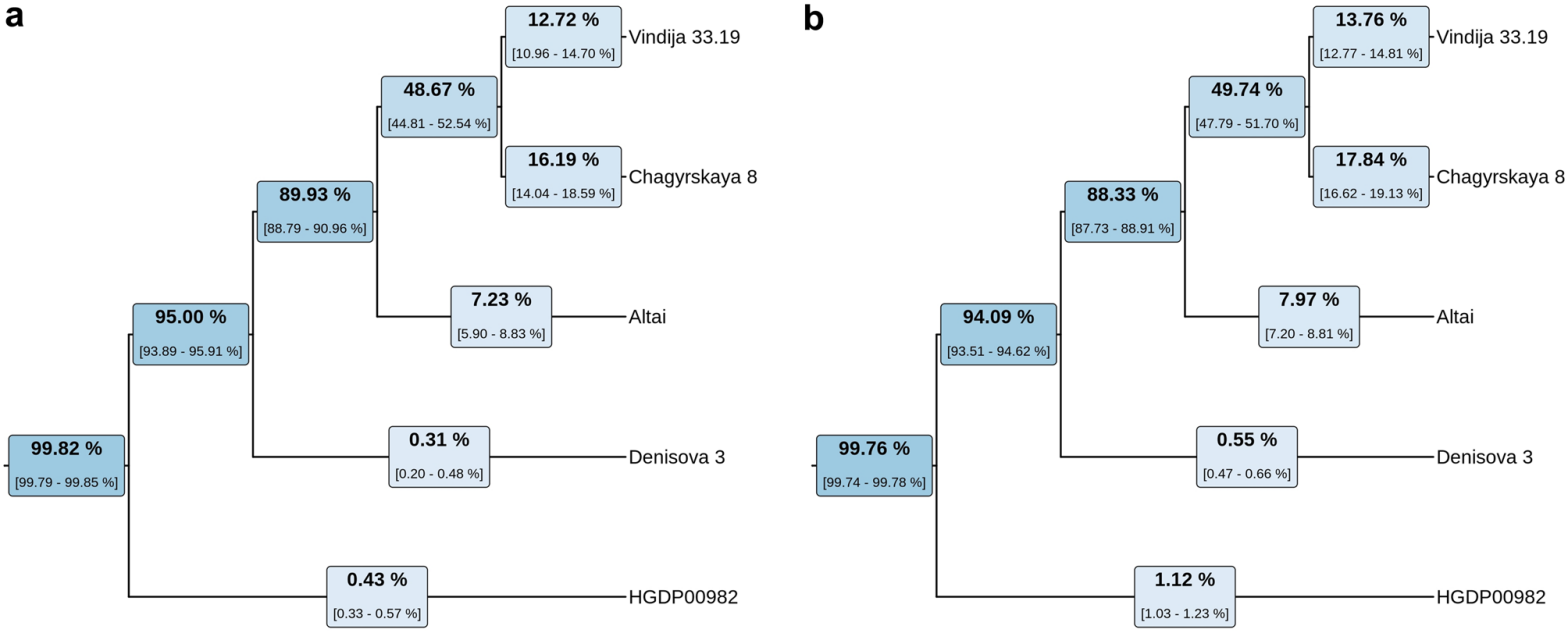
Autosomal derived allele sharing with the three high-coverage Neanderthals (*Vindija 33.19*, *Chagirskaya 8*, *Altai*), *Denisova 3* and present-day human (HGDP00982, Mbuti) for putatively deaminated (**a**) and total (**b**) *Mezmaiskaya 3* sequences (length ≥ 35, MAPQ ≥ 25). The 95% binomial confidence intervals are indicated in brackets.

We estimated the contamination rate for autosomal alignments of sequences generated from the *Mezmaiskaya 3* to be 2–4% using two different methods for sequences longer than 35 bp (see *SI*). We used nuclear sequences to evaluate the genetic proximity of *Mezmaiskaya 3* to high-coverage Neanderthal genomes (the *Altai*, *Chagyrskaya 8*, and *Vindija 33.19*), *Denisova 3* and present-day human (Mbuti, HGDP00982)^5,12,17,18^. We found that derived allele sharing of sample-specific variants^19^ is higher for Neanderthals (> 7%) than for *Denisova 3* and present-day African (< 1%), confirming the Neanderthal origin of *Mezmaiskaya 3*. We determined that *Mezmaiskaya 3* shares more sample-specific derived alleles with *Chagyrskaya 8* and *Vindija 33.19* than with the *Altai*. Based on the fraction of shared derived alleles, we can suggest that *Mezmaiskaya 3* is more closely related to *Chagyrskaya 8* (17% shared alleles) than to the late *Vindija 33.19* (13% shared alleles) (Fig. 4, Supplementary Table S4). Similar data was found by using D-statistic calculation. *Mezmaiskaya 3* shares significantly more derived alleles with *Vindija 33.19* and *Chagyrskaya 8* samples than with *Altai* (Z = − 5.562 and Z = − 3.514 correspondingly), and *Mezmaiskaya 3* was closer to *Mezmaiskaya 1* than to all other Neanderthals (Supplementary Figs. S6, S7).

### Neanderthal mitochondrial DNA variations and haplogrouping

Based on a reconstructed Bayesian phylogenetic tree of the coding region of mitochondrial genomes, as well as a complete set of mtDNA variations, we investigated the difference between phylogenetic groups of Neanderthals to detect the specific markers for each clade of the Neanderthal’s phylogenetic tree (Supplementary Table S13). Next, we proposed a hierarchical nomenclature for Neanderthal mtDNA haplogroups. In this nomenclature, the root clade is denoted NA (Neanderthal Ancestral). Nested clades are named by numerals and lowercase letters, such as in modern human mtDNA nomenclature^20^ (PhyloTree.org). Altogether, we labelled 18 distinct haplogroups characterized by specific mtDNA variants. Among them, we highlighted three major branches of Neanderthals corresponding to the relative age of the Neanderthal groups: Early (NE), Middle (NM), and Late (NL) Neanderthal clades (Fig. 3, Supplementary Fig. S10).

To facilitate the analysis of Neanderthal mitochondrial haplogroups, we used all available to date complete and partial Neanderthal sequence and developed a computational approach to automate the classification process by adapting HaploGrep 2^21^ to operate with the phylogenetic tree of the Neanderthal haplogroups (https://evolgenomics.org/haplogrep2-neanderthal).

### Phylogenetic re-evaluation of Neanderthals based on mtDNA haplogroups

In addition to analysing high-quality mtDNA sequences of Neanderthals (Fig. 3), the developed computational tool was used to perform a reanalysis of the available low-quality and short mitochondrial control region sequences (see SI, Supplementary Tables S14, S15). We found that the Gibraltar *Forbes Quarry* (*FQ*) individual belongs to the NM1 haplogroup together with the *Stajnia S5000* individual. Our data clarify the position of the *FQ* individual position within the Neanderthal clade^22^.

For the samples from northern Spain^23^, we found two different mtDNA haplogroups: NA1, the same as *Hohlenstein-Stadel* and *Valdegoba* individuals from Europe, and NM1, which is closest to the *Stajnia S5000* and Gibraltar *FQ* individuals. Samples from Chagyrskaya Cave, Altai^24^ were assigned to the late mitochondrial haplogroup NL (Supplementary Table S15, Supplementary Fig. S10). Three Neanderthal specimens from the Denisova Cave (E202, E213, and M65) were recently grouped with *Altai*, *Denisova 15*, *Mezmaiskaya 1,* and *Scladina I-4A*^24^, corresponding to the NE haplogroup. Actually, E202, E213, and M65 belong to a branch NA2a, rather than NE (Supplementary Table S14, Supplementary Fig. S10).

Using only the D-loop sequence available for the *Valdegoba* individual, we assigned it to the ancient NA1 haplogroup, confirming the previously reported data^25^, and further suggesting that this Neanderthal individual could be older than previously thought^26^. Additionally, the only D-loop 16258G variant presented in the available ultrashort *Altamura* mtDNA sequence (31 nt) enables us to assign this sequence to the exact haplogroup NL1 and confirm the late European lifetime of this Neanderthal (Supplementary Table S15, Supplementary Fig. S10). Interestingly, the *Teshik Tash*^27^ mtDNA control region has both NA1 (16242T) and NA2 (16156A) variants (Supplementary Table S15). Therefore, if the sequence is correct, we cannot exclude the existence of separate branches for this individual or different topologies of the NA clade of the Neanderthal mtDNA tree (Supplementary Fig. S10).

### Functional evolution of Neanderthal mitochondrial DNA sequences

We searched for potential functional variations in the mitochondrial genome fixed in all Neanderthals or in specific clades only (see *Supplementary Results*). First, we analyzed variants in the mtDNAs shared by all Neanderthals that are absent in present-day humans or have minor frequencies (Supplementary Fig. S11A, Supplementary Table S6). We found the 827G Neanderthal variant in the 12S ribosomal subunit RNA gene, which is rare in modern humans (MAF = 0.02) and absent in non-human primates and Denisovans. It was found to be located in the vertebrate mtDNA conservative site (PhyloP score = 4809^28^). It was also described as pathogenic with incomplete penetrance in modern human families with aminoglycoside-induced deafness^29,30^, and in combination with another mutation in the 12S ribosomal RNA gene, significantly enhances the penetrance of hearing loss^31^.

Next, we analysed the positions variable among the Neanderthals. To avoid potential sequence errors, we selected mtDNA variants occurring in at least two Neanderthals. We found such variants distributed through each of 13 protein-coding genes as well as ribosomal and transfer RNAs (see Supplementary Tables S6, S17, Supplementary Fig. S11B) with an expected significant enrichment of the variations in noncoding hypervariable regions (32%). A prevalence of transitions (90.8%) over transversions (9.2%) was found in Neanderthal mitochondria, as was observed in modern humans^32,33^.

In addition, we analysed variants that defined the major clades of Neanderthal mtDNA: the NA1 haplogroup, which corresponds to the most ancient Neanderthals; the NA2 haplogroup, which is the ancestor of the middle and late Neanderthals; and the NL haplogroup, which is the most recent group (Supplementary Fig. S10, Supplementary Table S17). The older group, NA1, bears the maximum number of tRNA variants among all Neanderthal clades (Supplementary Fig. S12, Supplementary Table S17). Branch NA2 has a large proportion of missense variants in protein-coding genes (27% of all NA2-specific variants), with the highest proportion of amino acid substitutions in both the *ATP6* (N/S = 3.0) and *ND1* (N/S = 2.0) genes (Supplementary Table S17).

We found no potentially functionally significant variants in the Neanderthal branch NM. The heteroplasmy variant at the 8839 position in the *Mezmaiskaya 3* mitochondrial sequence is located in the coding region of the mtDNA sequence and leads to both p.105Ala and p.105Thr amino acids in mitochondrial ATP synthase subunit 6. This variant occurs in different human mtDNA haplogroups, including African, Asian, and European populations, and is not pathogenic. The only nonsense variant in the Neanderthal mtDNA in the NL clade T3308C (p. M1*) in the *ND1* gene, although its pathogenicity has not been proven (see. *SI*).

## Discussion

Here, we present the complete mitochondrial DNA and partially genomic sequences for a new Neanderthal individual (*Mezmaiskaya 3*) found in the Eastern Micoquian layer 3 at Mezmaiskaya Cave. The mitochondrial genome of the *Mezmaiskaya 3* is closer to the *Mezmaiskaya 1* and *Stajnia S5000* Neanderthals associated with the geographically remote Eastern Micoquian context^3,4^ than to other contemporaneous Neanderthals of Western Europe associated with other MP cultural facies. *Mezmaiskaya 3* is another Neanderthal representative of European Micoquian culture; to date, only a few Neanderthal specimens of this cultural tradition from Europe have been sequenced^4,7,13^. The molecular genetic clock dated the *Mezmaiskaya 3* to 90–100 Kya. Nevertheless, our results indicate that both Mezmaiskaya individuals (*Mezmaiskaya 1* and *Mezmaiskaya 3*) lived at about the same time (or after) the *Stajnia S5000* MP/Micoquian Neanderthals from Central Europe. Based on mtDNA data, we can suggest that *Mezmaiskaya 3* was the last Neanderthal individual from the early Neanderthal’s branches (NE and NM), which were replaced during early MIS 3 (60–40 kya) by genetically distant late Neanderthal populations (branch NL).

To date, a common classification of Neanderthal mitogenomes has not been reported. We used the most comprehensive dataset of Neanderthal mitochondrial sequences to propose a hierarchical nomenclature for the Neanderthal mtDNA haplogroups, with the main clades denoted NA, NE, NM, and NL.

Ancient Neanderthal haplogroup NA was formed by several mitochondrial variants that are absent in each Denisovan individual but present in the total set of Neanderthals and therefore can be considered the most ancient Neanderthal Eve mitochondrial haplotype. NA is split into the NA1 and NA2 clades (Supplementary Fig. S10).

Currently known representatives of the NA1 inhabited Europe and represent a group of early Neanderthals. Their remains were found in the territory of modern Germany (*Hohlenstein-Stadel*) and Spain (*Valdegoba*). NA2 root branch is today represented by only one individual from Denisova Cave (D5276^24^), and this clade splits into branches NE and NA2a (Supplementary Fig. S10). The NA2a clade is represented by at least three Asian Neanderthals from the Denisova cave^24^. The NE mitochondrial clade includes both eastern (*Altai* and *Denisova 15*) and western (*Scladina I-4A*) early Neanderthals, and most likely, it was widespread in Neanderthals of MP Europe and Central Asia. It should be noted that there are eighteen mtDNA positions in which the European *Scladina I-4A* mtDNA is divergent from both Asian *Altai* and *Denisova 15*. Potentially, a separate clade (e.g., NE1) for Scladina-type mtDNA within the NE branch should be distinguished.

The NA2a splits further into the major NM and NL haplogroups (Supplementary Fig. S10). The NM individuals were only found in Europe, whereas the NL clade is spread in both the eastern (Chagirskaya, Okladnikov, and Denisova caves) and western parts of the Neanderthals areal.

European-specific haplogroup NM combines Neanderthals from the North Caucasus (*Mezmaiskaya 1*, *Mezmaiskaya 3*), Central Europe (*Stajnia S5000*), and the Iberian Peninsula (Gibraltar *Forbes Quarry* and Neanderthals from Galería de las Estatuas) (Supplementary Fig. S10) with the approximate individual ages of 100–130 kya, which correlates to the time of the warm interglacial stages MIS 5b-MIS 5e in Europe. Neanderthals of the NM haplogroup have only been found in Europe, and to date, no NM haplogroup individual has been found in the eastern part of Eurasia. The Iberian Peninsula specimens share common mtDNA variants with the *Stajnia S5000* but not with the *Mezmaiskaya 1* and *Mezmaiskaya 3* from the North Caucasus representatives of NM2 sub-branch, despite the phylogenic clustering of *Stajnia S5000 and Mezmaiskaya 1* and *Mezmaiskaya 3* into common NM haplogroup (Supplementary Fig. S10). Therefore, we can assume that there were two distant genetic lineages: one of them led to the formation of the NM2 haplogroup specific to the early Eastern Micoquian Neanderthal population in the Caucasus, and the second (NM1) represents the mixed haplogroup of culturally diverse Western–Central European Neanderthal populations.

Recently, the early European Neandertals from *Hohlenstein-Stadel* and *Scladina I-4A* were defined to be genetically closer to later Neandertals from Europe than to roughly contemporaneous early Neandertals from Altai based on nuclear genome, and unexpected deeply divergent mtDNA in the *Hohlenstein-Stadel* from other mtDNAs was revealed^34^. Our results further confirm the separate ancient lineage of mtDNA of the *Hohlenstein-Stadel*. It shares a mtDNA branch with at least three Neanderthals from two European caves (Valdegoba and Galería de las Estatuas). Additionally, we can hypothesize the next lineages of mtDNA evolution among European and Asian Neanderthals (Supplementary Fig. S10):

1. The European lineage of the oldest NA1 clade, which separated from the Neanderthal Eve or ancient Asian lineage during late MIS 9–MIS 8^25^, and later was completely replaced in Europe by mtDNA descendants of the Altai branch NA2.
2. The Asian lineage of the oldest NA2 clade represented by the early Altai Neanderthals during MIS 6–early MIS 5, which developed into the NE clade mostly represented by the later Altai and possibly by the European (*Scladina I-4a*) Neanderthals and then formed into the European-specific NM branch during late MIS 5 and both NL1 and NL2 sub-branches during early MIS 3.

In contrast to mtDNA data, the nuclear DNA data revealed that European Neanderthals, both early and later, share closer ancestry to each other, than to Asian Neanderthals^34^. Genome-wide data on the Denisovan and Neanderthal hybrid revealed that she shares more alleles with the late European Neanderthal than with the *Altai*^35^. Recently published data on the Gibraltar *Forbes Quarry* individual suggested that she was equally related to both *Vindija 33.19* and *Chagyrskaya 8*^22^. Our data suggest that *Mezmaiskaya 3* also shares significantly more derived alleles with *Vindija 33.19* and *Chagyrskaya* 8 than with *Altai*, with a slightly larger number of alleles shared between *Mezmaiskaya 3* and *Chaginskaya 8* (Fig. 4, Supplementary Table S4). Altogether, genomic and mitochondrial data suggest that both Neanderthals of mitochondrial NM haplogroup (*Mezmaiskaya 3* and *Forbes’ Quarry*) are representatives of a separate ancient population that formed a separate intermediate branch between the populations from the Chagirskaya Cave and the population of late European Neanderthals.

In total, our data show that most of the currently sequenced Neanderthals trace their mtDNA lineages from the Asian Neanderthal mtDNA branch NA2. The European lineage NA1 was found in only a few early European individuals and had not been preserved in the late populations. Our results also point to the lacunarity of the current data on Neanderthal mtDNA haplogroups. For example, the Neanderthal specimens representative of the NA2a, which is the ancestor of European Neanderthals of haplogroups NM and NL, are unknown yet in Europe. The late (NL) haplogroup is a sister clade to NM. In this clade, subbranches NL1 and NL2 only include European inhabitants, while representatives of the root NL branch were distributed through the Altai region.

The nomenclature presented here is based on currently available data, both high- and low-quality complete mitochondrial sequences and short D-loop sequences. Furthermore, it can be expanded and detailed upon reconstructing the new Neanderthal mitogenomes.

mtDNA sequences can be used not only for phylogenetic analysis but also for functional analysis and to search for potentially pathogenic variants. We found 14 missense variants as well as 9 variants in ribosomal or transfer RNA genes common in all tested Neanderthals. The fixed Neanderthals variant 827G in the 12S ribosomal subunit RNA gene was previously described as pathogenic with incomplete penetrance in modern human families with aminoglycoside-induced deafness^29,30^. We can tentatively speculate that in some groups of Neanderthals, if mushrooms containing aminoglycosides were included in their diet or due to a combination with another currently unknown mtDNA variant (as in modern humans haplogroup B^31^), there could be an increased frequency of deaf individuals in the population.

The total rate of nonsynonymous (NS) variants to synonymous (S) substitutions in the ancient Neanderthal NA clade (NS/S = 0.3) indicates the predominance of purifying selection of mtDNA coding region of root NA Neanderthal clade (Supplementary Table S17). The most significant mutations in Neanderthal mtDNA appear to have occurred during the splitting of the ancient NA branch into two highly distant clades, NA1 and NA2 (see Supplementary Fig. S10). NA1 haplogroup individuals shared the maximum number of tRNA variants, although none of them was predicted to be functionally significant (Supplementary Table S16). Variants in the NA2 clade possessed a significant number of amino acid variations both in the total coding region (NS/S = 0.64) and in several genes, including the *ATP6* and *ND1* genes (NS/S are 3.0 and 2.0 respectively) (Supplementary Table S17), which suggests a probably adaptive selection in the NA2 clade. *ATP6* and *ND1* in present-day human populations have statistically significant differences in nonsynonymous/synonymous substitutions between groups in different climates^36–38^. In total, we can suggest that both the *ATP6* and *ND1* genes have been subject to selection to the NA2 Neanderthal clade and may have played a role in the adaptation of Neanderthals to different climatic conditions or general climate changes. Due to the small number of individuals known today for this haplogroup and fragmentary information on their lifetime, we cannot exclude other evolution models in this ancient group of Neanderthals.

We found no potentially significant functional variants in the mtDNA of the middle (NM) and late (NL) Neanderthal branches. Nevertheless, we could not exclude the potentially pathogenic effect of T3308C variant and possible MELAS-like pathology^39^ in the late Neanderthal clade (see *SI*, Supplementary Fig. S13.

The late Neanderthal branch (NL) is formed by several mtDNA variations leading to amino acid substitutions in oxidative phosphorylation genes (*ND1*, *ND2* and *ND5*) and in the rRNA gene. Variation in oxidative phosphorylation genes can affect many body functions, including cell growth, signaling, and inflammation. In modern humans, mtDNA variants are thought to be important in human adaptation^40^. All major Neanderthal mitochondrial DNA haplogroups were found to have at least one variant with amino acid change (Supplementary Fig. 3). Based on human mtDNA annotation, we found no signs that these variations are severely deleterious in any Neanderthal mtDNA haplogroups. Therefore, it is unlikely that the disappearance of any of the Neanderthal groups is associated with pathogenic mutations in mtDNA. Nevertheless, we cannot ignore that the accumulation of numerous new potentially adaptive mtDNA variants in the NL branch can help one Neanderthal population adapt to new living conditions for resettlement at the end of the MP.

Previous mtDNA studies indicated that Neanderthals associated with geographically distant variants of Micoquian—*Stajnia S5000* and *Mezmaiskaya 1*—belong to a distinct mtDNA clade that separated early (during MIS 6, ~ 170–150 ka) from the mitochondrial genomes of early Neanderthals from Altai and from the early European Neanderthals^4^. Our new data on mtDNA sequencing for *Mezmayskaya 3* show that it belongs to the same population of early Micoquian Neanderthals as *Mezmaiskaya 1* and *Stajnia S5000*. However, we also defined that *Mezmayskaya 3* and *Mezmaiskaya 1* share the same NM2 haplogroup, which is likely specific to the Eastern Micoquian early Neanderthal population in the Caucasus, while *Stajnia S5000* belongs to NM1, alongside two Neanderthals from the Iberian Peninsula that are not associated with Micoquian (Fig. 1)^4^. This may indicate that gene flow from Western European Neanderthals affected the ancestry of *Stajnia S5000,* and NM1 probably represents a mixed haplogroup of culturally diverse Western–Central European Neanderthal populations.

This Micoquian early Neanderthal population that lived ~ 100–60 ka ago also shows a distant relationship to the later European Neanderthals that lived ~ 50–40 ka ago. A previous study of the *Mezmaiskaya 2* indicated a population turnover that occurred during the time between *Mezmaiskaya 1* and *Mezmaiskaya 2*^7^. All the Neanderthals from the Mezmaiskaya Cave are associated with either the earlier (*Mezmaiskaya 1* and *3*) or later (*Mezmaiskaya 2*) variants of the Eastern Micoquian industry^3^. Two opposite models for this turnover were proposed^7^: 1) the replacement of the earlier Micoquian Neanderthals in the Caucasus by a population related to Western European Neanderthals or 2) the replacement of Neanderthals in Western Europe by the later Micoquian population related to *Mezmaiskaya 2*.

Our results show that *Mezmaiskaya 2*, along with *Feldhofer 1* and *Feldhofer 2* from Central Europe, are also associated with the later Micoquian industries and share the same NL1 haplogroup with other late Neanderthals in Western–Central Europe. Based on the fact that *Stajnia S5000* provides much earlier evidence than *Mezmaiskaya 2* of gene flow from Western European Neanderthals, which has affected the ancestry of Micoquian populations in Central Europe, we assume that there was a replacement of the Eastern Micoquian Neanderthals by Western European Neanderthals, and that this replacement took place quite a long time. It began earlier (late MIS 5) in the northwestern region (*Stajnia S5000*) and spread later (early MIS 3) to the southeastern (*Mezmaiskaya 2*) areas of the Micoquian distribution in Central–Eastern Europe. Further work is necessary to more comprehensively explore whether this was the case.

## Materials and methods

A human tooth (*Mezmaiskaya 3*) reported in this paper was discovered in layer 3 within square N-19 (see SI). The morphological description and palaeogenetic data both attribute the *Mezmaiskaya 3* tooth to Neanderthals.

### DNA extraction and library preparation

An ancient tooth was used in the genetic analysis. DNA extraction was performed using 120 mg of tooth powder. All procedures were carried out in room facilities dedicated to analysing ancient DNA. DNA was extracted from tooth powder along with blank control, and DNA extract was converted into single-stranded DNA libraries^15^. The amplified and indexed libraries were sequenced on an Illumina HiSeq 2000/2500 system (for details, see SI).

### Genome sequences analysis

In total, we aligned 14,172,617 reads onto the human reference genome (GRCh37), 53,028 reads on the human mtDNA reference sequence (rCRS, NC_012920.1^41^), and 54 604 reads on the Neanderthal mtDNA reference sequence (NC_011137.1^42^) using BWA^43,44^ (see SI, Supplementary Tables S5, S5). We determined the sex of *Mezmaiskaya 3* by counting the number of sequences mapped to the X chromosome and autosomes (Supplementary Table S3).

### Mitochondrial DNA analysis

To reconstruct the mitochondrial genome of *Mezmaiskaya 3,* we used reads shorter than both 25 and 35 nucleotides. We called every mitochondrial base. To call poly-C stretches at position 310 and CA copy numbers at positions 521–524 of the human reference genome, we visually inspected each read to calculate the C and CAs numbers (for detail, see SI).

To explore how the individual genome of *Mezmaiskaya 3* relates to that of other hominins, a total of 28 Neanderthals, including the *Mezmaiskaya 3* sequence, 4 Denisovans^45–48^, a hominin from Sima de los Huesos (*Homo sapiens* heidelbergensis)^16^, 10 ancient humans, 53 present-day humans with the reference rCRS mt genome^49^ (including two Africans with the mitochondrial genome L0a1 haplogroup), and the chimpanzee (NC_001643) as an outgroup were used; multiple sequence alignments of mtDNA sequences were performed by MAFFT^50^. MEGAX was used for maximum parsimony tree construction, and BEAST2.0 was used to estimate the molecular age of *Mezmaiskaya 3* mtDNA (see SI, Supplementary Tables S11–S12)^51^.

Next, we analysed the total set of complete mitochondrial genome variants in 28 Neanderthal individuals and 4 Denisovans and Sima de los Huesos hominins to reveal the set of specific mtDNA markers for every clade of phylogenetic tree for Neanderthals (SI, Supplementary Table S13) and put them at the base of the Neanderthal mtDNA classification.

In total, we used a set of 54 Neanderthals to explore the diversity of Neanderthal mtDNA and search for functionally significant variants (see *SI*).

## Supplementary Information

Supplementary Information 1.

Supplementary Information 2.

Supplementary Information 3.

Supplementary Information 4.

## Data Availability

The complete mitochondrial sequence of *Mezmaiskaya 3* is deposited in GenBank (OM062614), and the raw data for each library are available in the NCBI SRA under accession number PRJNA765125. The tool for classification of the Neanderthal mitochondrial haplogroup is available at https://evolgenomics.org/haplogrep2-neanderthal.
