# Supplementary material for: Genomic analysis of a novel Neanderthal from Mezmaiskaya Cave provides insights into the genetic relationships of Middle Palaeolithic populations
Source: Sci Rep. 2022 Jul 29;12:13016. doi: 10.1038/s41598-022-16164-9 (PMC9338269; doi:10.1038/s41598-022-16164-9)
Supplement: Supplementary file 1 — Supplementary Information 1. [file 41598_2022_16164_MOESM1_ESM.docx]

**Genomic analysis of a novel Neanderthal from Mezmaiskaya Cave provides
insights into the genetic relationships of Middle Palaeolithic populations**

Tatiana V. Andreeva, Andrey D. Manakhov, Fedor E. Gusev, Anton D. Patrikeev, Lyubov V. Golovanova, Vladimir B. Doronichev, Ivan G. Shirobokov, Evgeny I. Rogaev

**SUPPLEMENTARY INFORMATION**

***The stratigraphical, spatial, and archaeological context of Mezmaiskaya 3***

Mezmaiskaya Cave is located in the Northwest Caucasus, Russia, at 1310 m asl in the Azish-Tau Ridge, which is a part of the Lago-Naki highland in the Kurdjips River basin (a tributary of the Belaya River, Kuban River basin), which is approximately 50 km south of the city of Maikop (Northwest Caucasus, Russia; Supplementary Figure S1). Deep snow covers the cave's surroundings in the winter (Supplementary Figure S1-II). The cave is formed in the Upper Jurassic massive dolomite limestone, is approximately 350 m^2^, and faces southwest (Supplementary Figure S1-I). L. Golovanova started excavations on the site in 1987, and approximately 100 m2 has subsequently been excavated (Supplementary Figure S2) to a maximum depth of over 6 m, reached in the lowermost stratum (layer 7) revealed a test pit^1^. To date, 6 Holocene strata, including a Neolithic layer dating to ca. 8–7.5 ka cal BP ^2^, and 20 Pleistocene strata have been identified over the excavation area. A hard calcite-carbonate breccia up to 10 cm thick separates the Holocene and Pleistocene strata, thus reflecting a break in sedimentation. The Pleistocene strata are most completely preserved towards the interior of the cave and include (from top to bottom) Epipalaeolithic layers 1–3 dating to ca. 17.5–12 ka cal BP, eroded layers 1–4, which deposited after to the Last Glacial Maximum (~25–19 ka), and six Upper Palaeolithic layers (1А1, 1А1/1А2, 1А2, 1В1, 1В2, and 1С) dating to ca. 40/39–23 ka cal BP, which are excavated deeper in the cave^3,4^ (Supplementary Figure S3). The Upper and Middle Palaeolithic strata are separated by stratum 1D. It lies atop the uppermost Middle Palaeolithic layer 2 and contains no archaeological findings or bones ^4^. The seven Middle Palaeolithic layers (2, 2A, 2B-1, 2B-2, 2B-3, 2B-4, and 3, from top to bottom; Figure 3-II) have been dated to ca. 70–40 ka BP using the electron spin resonance (ESR) method^5^, and they show the development over time of an Eastern Micoquian industry. The lowest Pleistocene layers (4–7), which were only excavated in a test pit, contained no archaeological material.

Layer 3 is characterized by a dark brown loam containing a dense accumulation of small (1–2 cm) and rounded limestone éboulis, with some medium-sized (2–5 cm) corroded limestone debris, rare larger limestone blocks, and calcite debris; the surface of this layer lies with a slight slope from east to west. Depth marks of layer 3, horizon 5 on quadrant N-19 vary from -205 to -207 in the western part, on the border with quadrant M-19, to -190 to -199 in the eastern part, on the border with quadrant O-19. The microprofiles running along the eastern (Supplementary Figure S3-II: A, B) and western (Supplementary Figure S3-II: C, D) parts of quadrant N-19 clearly show a quite dense and compact vertical distribution of faunal remains and artefacts recovered *in situ* in layer three on quadrant N-19, associated with the *Mezmaiskaya 3* tooth. Additionally, the excavation plan clearly demonstrate a hearth—spread mostly on quadrant M-19 and partially extending to quadrant N-19 (Supplementary Figure S3-I)—associated with *Mezmaiskaya 3* tooth discovery area..

The lowermost Middle Palaeolithic layers 2B4 and 3 at Mezmaiskaya have 14C results beyond the radiocarbon limit, reflected in the predominance of greater-than or near-background limit ages^6^, and therefore are consistent with the ESR chronology for these levels^5^, which suggests the mean ages range from 57 to 73 ka (including error m.argins). For layer three, three ESR dates obtained on herbivore teeth from excavation horizons 3 and 4, which corresponds to the upper occupational level in the layer, provide mean ages ranging from 60 to 73 ka BP_ESR/LU_ (Supplementary Figure S3-II). The lower part of layer three, which has an older age, can be dated approximately 85–100 ka, based on mean ages obtained using the branch-shortening estimates (95% HPD: ~65–140 ka and 95% HPD: ~57–114 ka, according to the fixed and estimated mutation rates, respectively) from the mtDNA genome of *Mezmaiskaya 1*, which was found at the base of this layer^7^. These results suggest a long depositional history of layer three in the interval from MIS 5c (96–87 ka) to MIS 4 (71–57 ka). Multidisciplinary research indicates that a relatively mild climate prevailed during the time of layer 3, and rodents inhabiting both forested and subalpine environments were found in this layer^1,8^.

The lithic assemblage from layer three at Mezmaiskaya Cave belongs to a regional variant of the Eastern Micoquian industry^1,9^. The assemblage is characterized by the predominance of flakes with parallel (~50%) and irregular (~25%) removals on the dorsal face, and the core reduction using mainly recurrent unidirectional (one-platform) and recurrent bidirectional (two-platform bipolar or orthogonal) cores. Among the prevailing tools made on flakes, simple side-scrapers predominate, and many of them have a thinning retouch made from a specially-prepared platform. Transverse, diagonal, and double scrapers are not characteristic of this industry. Bifacial and partial bifacial tools are numerous (~20% of total tools) and include tool types typical of the Eastern Micoquian industry, such as leaf-shaped bifacial points, wide and narrow small bifaces worked using the plano-convex method, and bifacial scrapers or scraper-knives. Convergent tools are also numerous (~20% of total tools) and variable, including elongated and short Mousterian points, short and small retouched points (most with thinning of the base of the tool), elongated and short convergent scrapers, and angled scrapers. End-scrapers made on flakes and flakes with fine retouch are also abundant. Besides stone tools, the assemblage also comprises an abundance of bone retouchers, bone flakes and chips, and retouched bone shaft fragments.

***Morphological description of Mezmaiskaya 3***

A tooth (*Mezmaiskaya 3*) reported in this paper was discovered in layer 3 within square N-19 (Figure 2B). In line 19, layer 3 had thickness up to 90 cm, and was excavated by nine arbitrary excavation horizons (Supplementary Figure S2). The tooth, which was recovered in horizon 5, was identified during the analysis of faunal material from water sieving. Cleghorn (2006) gave the first brief description of the Mezmaiskaya 3 tooth^10^. It was defined as a milk upper incisor with pronounced spatularity and a highly worn crown.

In the mesiodistal direction, the tooth's crown is crossed by an arched crack, with the arch oriented towards the vestibular side. Shallow cracks are also present in the enamel on the vestibular side of the crown—they are marked by black patches that are also present on the lingual side along the border of the crown. Approximately a third of the tooth root is preserved. The edges of the broken surface are uneven and smoothed in some parts. It is not possible to determine whether the absence of a part of the root is the result of resorption or ancient fracture (or both). The tooth crown is worn about half of the initial height, and a wide band of dentin and a pulp chamber are visible on the preserved surface. On the lingual side, the crown is worn stronger (the angle of inclination relative to the perpendicular to the vertical axis of the tooth is about 200). There is no evidence of pathologies on the tooth. It is known that teething in Neanderthals occurred at an earlier time, and the enamel thickness is less than in modern humans^11^; therefore, models for determining the age of modern humans are not suitable for determining the age of Neanderthals. However, the high degree of wear on the *Mezmaiskaya 3* crown suggests that the tooth has been functional for quite a long time.

The vestibular surface of the incisor is convex in both the vertical and mesiodistal directions. On the lingual side, the tooth has a shovel-like shape, and the mesial marginal ridge is more developed than the distal one. The lingual tubercle (*tuberculum dentale*) is moderately pronounced. The morphological features, such as a shovel shape and pronounced labial convexity, are often found on Neanderthal incisors^12^. While these features are usually more variable on milk incisors than on permanent incisors, the morphology of the *Mezmaiskaya 3* milk incisor is quite typically Neanderthal. According to Cleghorn's (2006) measurements made directly on the specimen^10^, the mesiodistal diameter of the crown is approximately 7.9 mm, and the buccolingual diameter is 5.7 mm (Figure 2). However, an underestimation of the mesiodistal diameter can not be excluded due to the strong wear of the crown.

Neanderthal teeth, including milk teeth, are on average slightly larger in size compared to the teeth of modern humans, while early Neanderthals have larger teeth compared to late Neanderthals ^13^. A comparison of the *Mezmaiskaya 3* tooth size with published data shows that the mesiodistal diameter in *Mezmaiskaya 3* fits into the range of variability typical to Neanderthals and exceeds the average values typical to both Upper Palaeolithic and living modern humans. The value of the buccolingual diameter is located in the border of values characteristic of Neanderthals and modern humans.

For a more detailed comparison of *Mezmayskaya 3* with other Neanderthal specimens, we used available published data on measurements of milk upper incisors from Middle Palaeolithic sites. In total, published data on tooth measurements for 23 individuals were used. Each individual is represented by a single tooth (when the measurements of both central incisors related to the same individual were presented, the measurements on the right incisor were taken into account). Unfortunately, data on Neanderthal milk incisors from the Central and Eastern European regions are not available. Data comparison shows that the value of mesiodistal diameter is likely underestimated at *Mezmaiskaya 3*, though probably only slightly. In terms of the ratio of mesiodistal and buccolingual diameters, *Mezmaiskaya 3* is noticeably different in the relatively large value of mesiodistal diameter from other Neanderthal specimens. In terms of the crown index, *Mezmaiskaya 3* is most similar to the later and geographically most distant Neanderthal specimens characterized by a noticeably larger crown size.

Morphological description and morphometric data both attribute the *Mezmaiskaya 3* tooth to Neanderthals. Among them, the northernmost Neanderthal samples from Europe—milk incisors of Spy VI, Engis 2, and Roc de Marsal—are most similar to the Mezmaiskaya tooth. Like *Mezmaiskaya 3*, all three Neanderthal specimens are characterized by a shovel-shaped crown with a convex vestibular surface.

***DNA extraction and library preparation***

The tooth powder was resuspended by vortexing in 0,5 M ethylene glycol tetraacetic acid (Sigma-Aldrich) with 0.2% SDS, 13 mmol DTT, and 0,6 mg/ml proteinase K, and the suspension was shaken at 56 °C for 2.5–3 hours. The remaining bone powder was pelleted by centrifugation in a bench-top centrifuge for 2 min at maximum speed. Ten volumes of binding buffer containing 6 M sodium perchlorate, 40% (vol/vol), and isopropanol, 60% (vol/vol), were added to the supernatant, and the mix was applied to MinElute Spin Columns (Qiagen) with centrifugation in a bench-top microcentrifuge for 30 sec at 6,000 rpm to avoid the loss of short fragments. After washing with 0.75 ml of Buffer PE, DNA was eluted in a final volume of 35 μl of Buffer EB (Qiagen) warmed to 56 °C. The blank controls were used during DNA extraction. One microlitre of DNA extract and blank controls were applied on a high-sensitivity DNA chip and tested on a Bioanalyzer 2100 (Agilent) (Supplementary Figure S4).

Subsequently, 5 or 8 μl of DNA extract was converted into single-stranded DNA libraries^14^. We performed DNA treatment with PreCR Mix (New England Biolabs) to repair DNA modifications before library preparation. DNA libraries prepared with nontreated DNA were used for authentication analysis of the resulting DNA fragments after genome sequencing. DNA extract prepared using PreCR Mix produced the best results in terms of the endogenous DNA content and ancient DNA modifications.

We did not analyse the libraries with qPCR before indexing because of the presence of adapter fragments in the library, which could contribute to qPCR results. Each library was amplified by 6–8 cycles with AccuPrime Pfx DNA polymerase (Invitrogen) and two library-specific uniquely barcoded primers. The PCR product was purified using the MinElute PCR purification kit and then size-selection to remove primer dimers that had formed during amplification as well as library molecules with very short (<20 bp) and very long (>100 bp) inserts was performed. Libraries were run on a 2% E-gel (Thermo Fisher Scientific) for 15–20 min. The gel cassette was opened, and the DNA fragment between 155 and 250 bp was excised. There were no visible DNA fragments larger than 100 bp in the negative control library; therefore, we did not use it further. Gel slices were dissolved in three volumes of QG buffer, and DNA was extracted using the QIAquick Gel Extraction Kit (Qiagen) according to the manufacturer’s protocol. The final DNA library was eluted in 20 μl EB buffer.

In total, two libraries were prepared for Neanderthal DNA samples one (MN_norep) with untreated DNA and one (MN_rep) with PreCR MIX-treated DNA.

The amplified and indexed libraries were sequenced either individually or in pools with other libraries on an Illumina HiSeq 2000/2500 system. Paired-end or single-read sequencing was carried out by 51 to 76 cycles (Supplementary Table 1). Most sequenced DNA fragments were short enough that single-end reads carry part of the adapters at their 3’ end. Therefore, paired reads only resulted in double sequencing of the same DNA molecule, making the experiment more expensive. Thus, single-sequencing reads are sufficient for sequencing such short DNA molecules.

***Processing of sequenced reads***

We used bcl2fastq v2.20 (Illumina) for demultiplexing and base calling of the sequencing data and AdapterRemoval v2.3.1 with the parameters ‘--collapse (for PE data) --mm 0.5 --trimns --trimqualities --trimwindows 1 --minquality 10 –minlength 25/35’ ^15^ for trimming the adapter sequences of raw reads. Among the trimmed sequences, reads shorter than 25 or 35 nucleotides were eliminated from further analyses. The results showed that reads of 25 nt are applicable for the reconstruction of mtDNA sequences for all tested libraries. In total, we aligned 14 172 617 trimmed reads of the *Mezmaiskaya 3* DNA libraries onto the human reference genome (GRCh37), 53 028 reads on the human mtDNA reference sequence (rCRS, NC_012920.1^16^), and 54 604 reads on the Neanderthal mtDNA reference sequence (NC_011137.1 ^17^) using BWA v0.7.17^18^ with parameters adopted for ancient DNA ‘-l 1024’, ‘-n 0.01’, and ‘-o 2’ ^19^ (Supplementary Table S2). Duplicates reads were marked using the Picard toolkit v2.22.2 ^20^ and were excluded from further analysis. We used mapDamage2 v2.2.1 software ^21^ with the parameters ‘--single-stranded --rescale’ to determine the per library postmortem degradation pattern of DNA and observed ancient DNA patterns (an increase in C to T and A to G changes towards the ends of the reads). Base substitutions at the ends of DNA sequences were related to the postmortem degradation pattern of ancient DNA (Supplementary Figure S5), and the presence of such damage supports the authenticity of the extracted DNA originating from ancient specimens. mapDamage2 was also used to rescale the base quality scores specific to the damage patterns of ancient DNA before variant calling.

***Mezmaiskaya 3* *sex estimation***

To determine the sex of the *Mezmaiskaya 3* specimen, we analysed short sequences realigned to the human reference genome (GRCh37) with a mapping quality of 25. Additionally, due to potential contamination with modern human DNA, the analysis was restricted to sequences with signs of deamination. We determined the sex of *Mezmaiskaya 3* by counting the number of sequences mapped to the X chromosome and autosomes (Supplementary Table S3). Because the coverage of the X chromosome was similar to that of the autosomes, we suppose that the *Mezmaiskaya 3* individual is female.

***Nuclear DNA Analyses***

We estimated nuclear genome contamination using two different approaches: by analysis of deamination patterns with AuthentiCT ^22^ and by estimating the proportion of observed modern human alleles^23^. We applied AuthentiCT only to sequences from untreated paired-end library using for analysis only those read pairs that have been merged by AdapterRemoval 2 to ensure availability of the complete sequence of the DNA fragment. To estimate contamination based on the presence of modern human alleles we followed the previously described approach^23^. At sites where four primates (*Pan troglodytes*, *Pongo abelii*, *Macaca mulatta,* and *Gorilla gorilla*) share the same allele, but the Mbuti (HGDP0456) allele is different from it and from both *Denisova 3* and *Altai* Neanderthal alleles (and excluding transitions)*,* we computed the proportion of alleles shared between *Mezmaiskaya 3* and Mbuti individual. We modeled it as a linear combination of allele sharing of the same Mbuti individual with modern human contaminant (French, HGDP00521) and with *Vindija 33.19* and estimated contamination from this model. Finally, we estimated the 95% confidence intervals (CI) using binomial distribution, as described previously^23^.

For sequences longer than 35 bp and MQ at least 25 in highly mappable regions of the human genome, we estimated the contamination rate to be 3.99± 0.27% using the deamination pattern analysis approach. However, it was reported to overestimate the contamination rate at low levels of contamination. In line with this, we found lower contamination rate of 2.1% (CI 1.74 – 2.49%) using the analysis of allele sharing with a modern human.

For the analysis of the *Mezmaiskaya 3* genomic sequences, we used a minimum read length of 35 bp. To avoid a possible reference bias we used minimum mapping quality of 25. Further, only putatively deaminated reads were kept for downstream analysis (those with C-to-T substitutions in the first or last three nucleotides). For lineage attribution, we quantified the fraction of derived alleles^24^ shared between *Mezmaiskaya 3* and three high-coverage Neanderthals (*Altai, Vindija 33.19,* and *Chagyrskaya 8*), Denisovan, and modern human of sub-Saharan origin (HGDP00982)^25–28^. Within these five genomes, we identified sample-specific derived alleles (present only in one of the samples but not in the other four), excluding C-to-T and G-to-A variants. We only considered positions where *Pan troglodytes*, *Pongo abelii*, *Macaca mulatta,* and *Gorilla gorilla* reference genomes (CHIMP2.1.4, PPYG2, MMUL_1, and gorGor3.1) have the same allele in EPO 6-way primate alignment (denoting this allele as ancestral)^29–31^ and within the highly mappable regions of the human genome (map35_100)^25^. Additionally, we excluded positions where other alleles were observed in *Mezmaiskaya 3*. To estimate allele sharing of Mezmaiskaya 3 with a particular branch of high-coverage samples, we computed the number of positions with branch-specific variants (present in all samples of the branch and in no other samples) covered by *Mezmaiskaya 3* autosomal alignments and the number of such positions where the same derived allele is observed in *Mezmaiskaya 3* fragments^32^.

Additionally, we evaluated the genetic proximity of Mezmaiskaya 3 to other Neanderthals using D-statistics that measures the fraction of derived allele-sharing between the genomes and can be used as a genetic distance estimate. We used three high-coverage genomes: the earliest known Neanderthal *Altai* individual from Denisova cave^25^; *Vindija 33.19* from Croatia^28^, and Neanderthal from Chagyrskaya Cave^26^. We also used five low-coverage genomes: *Goyet Q56-1* and *Spy94a* from neighboring caves in Belgium; *Les Cottes* neanderthal from Les Cottes cave in France, *Mezmaiskaya 1* and *Mezmaiskaya 2* from Mezmaiskaya cave^7,25^. We applied the same reads and variants filtering procedure as we used for nuclear genome-based linage assignment analysis. Pseudohaploid genotypes were called using the pipeline suggested by and tool provided by Stephan Schiffels (https://github.com/stschiff/sequenceTools) that is currently widely used for low-coverage pseudohaploid variants calling. The pipeline combines samtools mpileup calling (with a filter of minimum base quality of 30 and mapping quality of 25 (--min-BQ and --min-MQ) applied) and sequenceTools pileupCaller for calling genotypes by randomly choosing one allele from each site where there was read coverage. Datasets merging with consecutive removal of non-biallelic variants was performed using BCFtools^33^ and PLINK^34^. Derived alleles sharing analysis via D-statistics calculations was performed using AdmixTools qpDstat program with the integrated jackknife block method for statistical significance computation^35^. The same pipeline was applied to both *Mezmaiskaya 3* data and Neanderthal data obtained from previous publications.

We found that *Mezmaiskaya 3* shares significantly more derived alleles with *Vindija 33.19* and *Chagyrskaya 8* samples than with *Altai* Neanderthal (Z = -5.561 and Z = -4.548 correspondingly). Further, we detected no statistically significant differences in derived allele sharing of *Mezmaysksaya 3* when compared to *Chagyrskaya* and *Vindija 33.19* genomes (D±se = 0.03±0.03355, Z = 0.893) (Supplementary Figure S6).

Next, to clarify the proximity of *Mezmaiskaya 3* to Neanderthals from distinct time periods, we analyzed the genetic distances from *Mezmaiskaya 3* to *Mezmaiskaya 1* and *Chagyrskaya 8*, as well as *Vindija33.19* and *Mezmaiskaya 2*. We observed a statistically significant signal of *Mezmaiskaya 3* higher proximity to *Mezmaiskaya 1* than to *Chagyrskaya 8* (Z = -2.343) (Supplementary Figure S7).

Finally, to determine which of the two Neanderthals genomes was older, we used D-statistics analysis to compare *Mezmaiskaya 1* and *Mezmaiskaya 3* to *Vindija 3.19* and *Altai*. We found no significantly differences when compared *Mezmaiskaya 1* and *Mezmaiskaya 3* to *Vindija 3.19* both for desaminated (D±se = 0.072±0.047, Z = 1.52) and total reads (D±se = 0.73±0.020, Z = 0.73).

### *Reconstruction of the mtDNA genome*

To reconstruct the mitochondrial genome of *Mezmaiskaya 3,* we first assessed the contamination rate . We used reads shorter than both 25 and 35 nucleotides. To estimate present-day human contamination, we used different approaches. *Mezmaiskaya 3* sequences were realigned to the revised Cambridge Reference Sequence (rCRS, NC_012920.1) and the Neanderthal mitochondrial genome reference sequence (NC_011137.1) ^17^ (Supplementary Table S5). We tested 42 present-day human-specific mutation positions at which 99,9% of 51 836 GenBank mitochondrial DNA sequences (greater than 15.4 kbp, February 05, 2021) differed from that of 28 Neanderthals (see Supplementary Table S6). For both the repaired PreCR MIX and native DNA libraries, we computed the proportion of sequences that carried both modern human and Neanderthal alleles. Modern human variants were observed with minor frequencies in the deaminated DNA fragments and the total sequences. In total, human mitochondrial DNA contamination did not exceed 1,6% for the deaminated sequences and 6,1% for the total mitochondrial mapped sequences (Supplementary Table S7).

Estimated contamination in the mtDNA sequences using a Schmutzi algorithm^36^ was 2% for the nonrepaired library.

Based on the low level of present-day human contamination, we used a cut-off of 25 bp for sequences to mtDNA genome reconstruction. To minimize the potential bias of using the human reference mitochondrial genome, we mapped the *Mezmaiskaya 3* reads to the reference Neanderthal mtDNA genome (NC_011137.1). A total of 1 092 209 reads were aligned to the Neanderthal mitochondrial genome with a resulting coverage of 91.25x (MQ>=25) (Supplementary Table S5). To reconstruct the mitochondrial genome of *Mezmaiskaya 3*, we called every mitochondrial base. To call poly-C stretches at position 310 and CA copy numbers at positions 521–524 of the human reference genome, we visually inspected each read to calculate the C and CAs numbers. In total, at least 83% of fragments carried an identical base at every mitochondrial genome position except position 8839 of the human reference genome. We also identified four *Mezmaiskaya 3*-specific variants rare in human populations and absent in other Neanderthals (Supplementary Table S8, Supplementary Figure S8). A total of 67.86% of sequences carried the G variant at position 8839, and 32.14% carried the A variant (Supplementary Table S9). The allele frequency of the unique Neanderthal *Mezmaiskaya 3* individual 8839A allele in the human population was low (0,127%). The 8839G variant was found to be in an evolutionarily conserved mtDNA site (Supplementary Table S8). Taking into account the ratio of the reads with both alleles (~1:2), we can exclude a nuclear mitochondrial sequence (NUMTs) contamination. Based on the low human mitochondrial DNA contamination and the level of C to T substitution in the *Mezmaiskaya 3* specimen, we can postulate the heteroplasmy status (G/A) at the 8839 position in the *Mezmaiskaya 3* mitochondrial sequence. Minor frequency variants at other mitochondrial DNA positions could represent postmortem mutations in mtDNA or contamination by humans or the mtDNA of another species.

***mtDNA phylogenetic analysis***

To explore how the *Mezmaiskaya 3* individual mitochondrial genome relates to that of other hominins, high-quality mtDNA sequences of 28 Neanderthals, 4 Denisovans^37–40^, a hominin from Sima de los Huesos (*Homo sapiens* heidelbergensis)^41^, 10 ancient humans, 53 present-day humans with the reference rCRS mt genome^42^, and the chimpanzee (NC_001643) mitochondrial genome as an outgroup, were used. Multiple sequence alignments of mtDNA coding sequences were performed by MAFFT^43^. MEGAX was used for maximum parsimony tree construction using the subtree–pruning–regrafting algorithm, and 1000 bootstrap repetitions were carried out to evaluate the support for each branch. We restricted our analysis to only the coding region of mtDNA (positions 577–16023 of the rCRS sequence). If any mitochondrial coding region position was missed in at least one sequence, we removed this position from all genomes. This maximum parsimony tree supports that *Mezmaiskaya 3* has a Neanderthal-like mitochondrial genome and indicates the close relationship of *Mezmaiskaya 3* to the *Mezmaiskaya 1* and *Stajnia S5000* mitochondrial genomes (Supplementary Figure S9).

The pairwise differences between a complete mtDNA sequence of *Mezmaiskaya 3* and other Neanderthals revealed that *Mezmaiskaya 3* mtDNA has the fewest differences from early Neanderthals *Mezmaiskaya 1* and *Stajnia S5000*. In total, the complete mitochondrial genome of *Mezmaiskaya 3* differed by 20 substitutions from *Mezmaiskaya 1* and by 26 substitutions from *Stajnia S5000* (Supplementary Table S10).

***Dating the mtDNA genome of Mezmaiskaya 3***

BEAST2.0 was used to estimate the molecular age of *Mezmaiskaya 3* mtDNA^44^. We used the coding region of mitochondrial genomes for Neanderthals and ancient humans whose remains were directly radiocarbon-dated (Supplementary Table 13), as well as present-day human mitochondrial genomes^42^, including two randomly selected modern human mitochondrial genomes with the L01a haplogroup, which is close to the most recent common ancestor for all human mitochondrial DNA lineages (GenBank ID: KJ185432 and KJ186009). Mitochondrial sequences of the hominin from Sima de los Huesos were excluded from the analysis due to many undetermined mtDNA genome positions. We tested several separated datasets, which included or excluded sequences with large gaps or missing data (Hohlenstein-Stadel and GoyetQ57–1), as well as modern humans with the L01a mitochondrial haplogroup. We found no significant differences for all tested datasets. Thus, for consistency with previously published data^45,46^, the final dataset included the coding region of the same mitochondrial genomes: 26 Neanderthals and 10 ancient humans whose remains were directly radiocarbon-dated (Supplementary Table S11), as well as 53 present-day human mitochondrial genomes^42^.

The best-fitting substitution model chosen by jModelTest v. 2.1.10^47^ for the final dataset was a general time-reversible (GTR) model with a gamma-distributed rate heterogeneity (GTR+G). We fixed the mutation rate at 1.57x10^-8^ substitutions per site per year for the coding region of the mtDNA genome sequence^48^. Strict and uncorrelated lognormal relaxed molecular clocks were tested with a constant size and Bayesian skyline tree priors. For directly radiocarbon-dated individuals, we used radiocarbon-calibrated dates as uniform priors for the tip dates of their mitochondrial sequences. For undated individuals, we set an initial value of 50 kya and a uniform prior from 30 to 200 kya for Neanderthals and from 30 to 300 kya for Denisovans. The *Denisova 3* date prior was set between 30 and 100 kya^28,49^. The tip dates of modern individual samples were set to 0, and a monophyletic group of modern humans uniform prior to 50 kya to infinity for the most recent common ancestor of modern humans was created. The second monophyletic group uniform prior to 100 kya to infinity was set for Neanderthals. For the *Sima de Los Huesos* individual, we set the prior age to a range from 260 to 780 kya^41,50^. We used three independent Markov chain Monte Carlo (MCMC) runs of 30,000,000 iterations, sampling parameter values, and trees every 1000 iterations. The first 10% of the steps of the MCMC were discarded as burn-in.

Stepping stones and path sampling were used to compare the models. The best fit of the data with the Strict clock and Bayesian skyline tree priors was used for four independent MCMC runs, with 75,000,000 iterations each. We combined the results with LogCombiner and used 10% of the iterations as burn-in.

The resulting BEAST tree was visualized using FigTree v1.4.4^51^. Based on the length of the *Mezmaiskaya 3* branch, we estimated the *Mezmaiskaya 3* individual date to be 96.7 Kya (95% highest posterior density interval from 59 to 134 kya) (Supplementary Table S12).

The present analysis assumes that the mutation rate in Neanderthals and Denisovans is the same as in modern humans and cannot detect back-mutations or multiple substitutions that occur at the same position of the mitochondrial genome in different branches. Therefore, our molecular age estimations of *Mezmaiskaya 3* mitochondrial DNA are not absolute ages.

***Genetic variation of the mitochondrial sequences of Neanderthals and haplogroup estimates***

We analysed the total set of complete mitochondrial genome variants in 28 Neanderthal individuals, 4 Denisovans, and Sima de los Huesos hominins. Using as a basis the reconstructed Bayesian phylogenetic tree of coding region mitochondrial genomes, we analysed complete mtDNA sequences for each branch of the tree. We revealed the set of specific mtDNA markers for every clade of the phylogenetic tree for Neanderthals and named every branch (Supplementary Table S13).

To facilitate the analysis of Neanderthal mitochondrial haplogroups, we developed a computational approach to automate the process of classification. HaploGrep 2^52^ is a widely used tool for haplogroup analysis of modern human mitochondrial sequences. We adapted it to operate on the Neanderthal haplogroup phylogenetic tree to allow for fast classification using complete or partial Neanderthal mitochondrial genomes. Common present-day mitochondrial variants were also used to discriminate between modern human and Neanderthal sequences. The tool is available at <https://evolgenomics.org/haplogrep2-neanderthal>.

Initially, we only used complete mtDNA sequences used for Bayesian tree reconstruction, tool testing, and mtDNA haplogroup estimation. Additionally, we retrieved the recently published mitochondrial sequences for the *Riparo Biron* Neanderthal^46^, Gibraltar *Forbes Quarry* specimen^53^, 15 sediment specimens with the highest number of unique Neanderthal mtDNA fragments in the sequenced libraries from Galería de las Estatuas, Denisova Cave, and Chagyrskaya Cave^54^, and 3 coding region sequences from Denisova Cave^55^.The set of seven mitochondrial DNA control region sequences was also used in the analysis.

We assign a separate clade for the *Stajna S5000*. Both variants assigned to the haplogroups NM1 are present not only in Gibraltar *Forbes Quarry* and Galeria de Estatuas, but also in *Stajnia S5000* individual, therefore, it is unlikely that they are errors and can be used for haplogrouping. At the same time, there are no variants of 6040G and 16139A in any Galeria de Estatuas and *Forbes Quarry* Neanderthals. We cannot exclude a slightly different structure of this branch, that can be updated, if a new data becomes available.

Mitochondrial haplogroups were estimated for each of 54 available Neanderthal mtDNA sequences (see Supplementary Figure S10). In total, the analysis of individual mtDNA variants with the tool can be especially useful if the material is of poor quality (for example, from sediments) and it is not possible to obtain a complete mtDNA sequence.

***Phylogenetic re-evaluation of Neanderthals based on mtDNA haplogroups***

We used the developed computational tool to reanalyse the available Neanderthal mitochondrial sequences (Supplementary Tables S14, S15). First, we determined the haplogroups for the sequences of the two Neanderthal individuals from Riparo Broion, Italy^46^ and the Gibraltar *Forbes Quarry* individual^53^. The *Riparo Broion* mitochondrial sequence shares the total set of NL1a2b haplogroup markers and belongs to the late NL1a2b mtDNA branch. The *Forbes Quarry* (FQ) individual has both NE and NM (NM1) mtDNA haplogroup markers and no NL variants (except for the 5262G variant, which is likely an individual FQ variant) and thus belongs to NM1 along with the *Stajnia S5000* individual (Supplementary Tables S15, Supplementary Figure 8).

Next, using our haplogroup tool, we determined the Neanderthal mtDNA haplogroups for several recently generated Neanderthal mitochondrial sequences from northern Spain and Altai (Supplementary Tables S14)^54,55^. Two different mitochondrial haplogroups were found in specimens from northern Spain. The first one (NA1) is consistent with that of the *Hohlenstein-Stadel* and *Valdegoba* individuals from Europe. The second mitochondrial haplogroup is the Middle Neanderthal clade NM1, which is closest to the *Stajnia S5000* and *Gibraltar Forbes Quarry* individuals. All samples from Chagyrskaya Cave were assigned to the late NL mitochondrial haplogroup. Three Neanderthal specimens from Denisova Cave (E202, E213 and M65), which were recently grouped with *Altai*, *Denisova 15*, *Mezmaiskaya 1,* and *Scladina I-4A* individuals^55^, actually belong to the haplogroup NA2a rather than NE (corresponding to *Altai*, *Denisova 15,* and *Scladina I-4A*) or NM2 (corresponding to *Mezmaiskaya 1*) (Supplementary Table S14, Supplementary Figure 8).

In addition, we analysed the short mitochondrial control region sequences (D-loop) of seven Neanderthal individuals from GenBank (Supplementary Table S15).

Most of the analysed D-loop sequences (*Altamura* and *Monty Lessini* from Italy, *Vindija 75* from Croatia, and *El Sidron 441* and *El Sidron 1351e* from Spain) belong to European haplogroup NL1 with the common 16258G variant, which is absent in all earlier Neanderthals groups (NA, NE, and NM).

In total, three control region variants (16242T, 16294T, 16304C) refer to the *Valdegoba* to ancient (NA1) Neanderthal mitochondrial haplogroup (Supplementary Table S15, Supplementary Figure 8). Therefore, based on the control region sequence, we can assume that *Valdegoba* mitochondrial DNA could be older than previously thought^56^, and it seems to be more closely related to the mtDNA sequence from ancient *Hohlenstein-Stadel* individuals than to other European Neanderthals.

The *Teshik Tash* control region sequence seems to belong to the early Neanderthal haplogroups NA (Supplementary Table S15, Supplementary Figure 8), which confirms the closer relationship to the mtDNA sequence with the *Scladina I-4A* individual than with the *Okladnikov 2* individual^57^. It should be noted that the *Teshik Tash* mitochondrial DNA control region has both NA1 (16242T) and NA2 (16156A) variants. Therefore, we cannot exclude the existence of separate branches for the *Teshik Tash* individual or different topologies of the whole NA clade of the Neanderthal mitochondrial DNA tree.

***Genetic similarity and diversity of the mitochondrial sequences of Neanderthals and mitochondrial haplogroup estimates***

To determine the mitochondrial variants common for all Neanderthals, we analysed the set of 29 high-quality complete mitochondrial genomes, the same ones that were used for phylogenetic analysis, including the *Mezmaiskaya 3* mtDNA sequence. A total of 77 mtDNA positions shared Neanderthals-specific alleles that are rare (MAF<10%) or absent in modern humans, 4 of which were exclusive to Neanderthals (Supplementary Table S6). The majority of these variants (79%) were present in the protein-coding region, with 18% of the total variants being missense variants (Supplementary Figure S11A). These coding region variants in modern humans were described in patients with several pathologies, including Leigh syndrome, Kearns–Sayre syndrome, MELAS syndrome, and Leber hereditary optic neuropathy^58^, but none of them was found to be a strict pathogenic variant.

Outside of the protein-coding genes, six variants shared by all Neanderthals were found in ribosomal RNA genes, and three were found in transfer RNA genes. Fixed in Neanderthal variant 827G in the 12S ribosomal subunit, the RNA gene is rare in modern humans (MAF=0.02) and absent in Denisovans and primates. It was described as pathogenic in modern human families with aminoglycoside-induced deafness^59,60^. This variant is common among carriers of human mitochondrial haplogroup B (MAF>0,8), and it has been shown that another mutation in the 12S ribosomal subunit RNA gene (1555G) increases the risk of deafness in haplogroup B^61^. In general, these data suggest a high risk of developing deafness in carriers of this mutation. No other common Neanderthal variants in rRNA and tRNA gene variants were predicted to be disruptive or damaging by using HmtVar Pathogenicity Prediction and MitoTip Ranking^62–64^ (Supplementary Table S16).

Next, we used a set of all available mitochondrial sequences of Neanderthals to explore the diversity of Neanderthal mtDNA and search for functionally significant variants, including recently published mitochondrial sequences for the Riparo Broion^46^ and Gibraltar Forbes Quarry specimens^53^, 15 sediment specimens with the highest number of unique Neanderthal mtDNA fragments in the sequenced library from Galería de las Estatuas, Denisova Cave and Chagyrskaya Cave^54^, and seven mitochondrial control region sequences whose haplogroups were identified (see Supplementary Figure 8). We excluded three coding sequences from Denisova Cave (E202, E213, and M65 from Zavala et al., 2021) from this analysis due to the high contamination by mtDNA of other species and potential errors in variant calling at evolutionarily conserved sites of mtDNA. For the low-quality mitochondrial genomes from the Riparo Broion, Italy^46^ and Gibraltar Forbes Quarry^53^ Neanderthal individuals and specimens from^54^, only diagnostic positions for Neanderthal mitochondrial haplogroups were assessed (Supplementary Table 13) to prevent genotyping errors.

In total, we detected 306 polymorphic sites, including insertions, deletions and homopolymeric site variants, among the total set of 51 Neanderthals. Sixteen homopolymeric insertion/deletion variants were excluded from further analysis due to the inability to determine the exact state of these positions in most samples. To avoid potential sequencing errors, only mtDNA variants occurring in at least two Neanderthal individuals were used to analyse the genetic diversity, and in total, 168 such variants were found in Neanderthals (Supplementary Table S6).

The distribution of polymorphic Neanderthal mtDNA variants shared by at least two individuals is represented in Supplementary Figure S11B. These polymorphic positions were distributed through each of 13 protein-coding genes as well as ribosomal and transfer RNA genes (Supplementary Table S6, Supplementary Table S12), with a significant amount in the noncoding hypervariable region (32%). The maximum number of coding region polymorphic sites, as well as missense variants, were found in the *ND5* (19 positions, 5 missense) and *ND1* (15 positions, 6 missense) genes. A higher prevalence of transitions (90.8%) than transversions (9,2%) was found in the Neanderthal mitochondrion, which is also observed in modern humans^65,66^. More than half of these variants (56%) were in protein-coding regions, and the maximum transition number was observed in the *CYTB* gene.

Next, we analysed the variants leading to each main clade of Neanderthal mitochondrial DNA: the NA1 haplogroup, which is related to early Neanderthals; the NA2 haplogroup, which is the ancestor of the middle Neanderthal haplogroup NM; and the NL Neanderthal haplogroup, which is the most recent (Supplementary Figure S10, Supplementary Table S13).

Both ancient Neanderthal clades (NA1 and NA2) differ from the root NA clade by a comparable number of mtDNA variants (41 and 32). An older group, NA1, which includes the European Neanderthals from Germany and Spain, bears the maximum number of tRNA variants among all Neanderthal clades (Supplementary Table S13, Supplementary Figure S10). The branch NA2, which is ancestral for most identified Neanderthal individuals, does not have a specific tRNA variant but contains a large proportion of missense variants in protein-coding genes (27% of all NA2-specific variants). The *ATP6* gene has the highest rate of amino acid changes in the NA2 clade. It should be noted that ATP6 has the highest amino acid sequence variation of any modern human mtDNA gene, and similar to that in the NA2 Neanderthal mtDNA branch, the number of nonsynonymous substitutions in the *ATP6* gene is greater than the number of synonymous substitutions in Asian and European mtDNA haplogroups but not in African haplogroups^67–69^. The number of missense variants in the *ND1* gene is also greater than the number of synonymous substitutions in the NA2 mtDNA clade of Neanderthals. For modern humans, a statistically significant difference in synonymous/nonsynonymous substitutions between groups in different climates was shown^67^. In total, we can suggest that both the *ATP6* and *ND1* genes were subjected to selection in NA2 Neanderthals and may have played a role in the adaptation of Neanderthals to climate change.

Two additional NA2 clade missense mutations in the *ND1* gene are present in the late Neanderthal haplogroup NL (Supplementary Figure 8). The only nonsense variant in all available Neanderthal mtDNAs is the T3308C (p. M1*) variant in the ND1 gene in the NL clade. An alternate start codon methionine of the *ND1* gene is located at amino acid position 3, suggesting that this mutation is not a true loss-of-function variant. Nevertheless, there is some evidence in modern humans that T3308C could be pathogenic in exact mtDNA backgrounds^70^. The Neanderthal haplogroup NL bears the 3308C allele on a unique background (Supplementary Figure S13); therefore, we could not exclude its potentially pathogenic effect and possible MELAS-like pathology (mitochondrial encephalomyopathy, lactic acidosis, and stroke-like episodes) in the late Neanderthal clade.

Finally, in the middle Neanderthal branch NM, the only potentially significant variant is the *ND4* missense mutation T12011C (p. Ser418Pro) (Supplementary Table S12). However, data are not available on its functional or pathogenic role in present-day humans.

***Cultural patterns of the Middle Palaeolithic Neanderthals***

The combination of chronological, paleogenetic, and archaeological data has a great significance for understanding both the population history of Neanderthals and the development of their cultural contexts^45^. Researchers in Western Europe propose different cultural, functional, and chronological interpretations for the named Middle Palaeolithic facies or cultures defined by F. Bordes^71^, such as the Ferrassie, Quina, Typical Mousterian, Denticulate, Mousterian and Mousterian of Acheulean Tradition (MTA), and for the MP technological variants or technocomplexes defined over the past forty years by various scholars, such as several types of Levallois, Laminar (Blade), Discoidal, Quina, and Bifacial technologies (see review in^72^. The chronological and geographic patterning of the Bordian Mousterian facies and the MP lithic technologies shows that some of them are quite limited in time and space, such as the Quina, MTA, and Denticulate facies, and Quina technology, and these may represent 'archaeological cultures', developed by some Neanderthal populations^72^.

It is also worth noting that all of the aforementioned culture-meaningful archaeological entities occur late in the Middle Palaeolithic, being mostly restricted to the period from late MIS 5 through early MIS 3 in Western Europe. This indicates that these cultural entities are associated with late Neanderthal populations and suggests that the origin of these MP cultures may be the result of population clustering among late Neanderthal groups. Without respective palaeogenetic data, the ultimate meaning of these defined MP archaeological units remains unclear. However, little is known whether the genetic relationship among late Neanderthal populations are correlated to their cultural proximity^7^.

On the contrary, the Typical Mousterian facies and the Levallois and Discoidal technologies are widely distributed through the entire Middle Palaeolithic period (between ~300 and 40 ka) and comprise a great variability of geographically distant lithic assemblages. These MP archaeological units broadly reflect only the common 'Middle Palaeolithic' styles of stoneknapping and toolmaking^72^.

Among the defined MP archaeological units, the Micoquian (or Eastern Micoquian, defined also *Keilmessergruppen,* KMG) is the broadest and longest cultural entity or technocomplex of the MP Neanderthals. In a broader perspective, research reveals strong similarities among the lithic assemblages from the Central European Micoquian, the Eastern Micoquian, and Chagyrskaya Cave in Altai^73^. The common features of these Micoquian Neanderthal groups bifacial and partial bifacial tools that include leaf-shaped bifacial points, wide and narrow small bifaces worked using the plano-convex method, and bifacial scrapers or scraper-knives^45,74^. These characteristics are typical of high mobility with recurrent short-term or seasonal occupations and land-use strategies developed for seasonal acquisition of high-migratory, cold-adapted, ungulate animals. The high mobility patterns of Micoquian Neanderthals across the Northern and Eastern European plains with steppe/forest–steppe cold environments could explain the widespread distribution and prolonged production of specific Micoquian stone tools compared to the other MP cultural entities. Studies indicate that the number and diversity of Micoquian sites increased during late MIS 5 – MIS 3^75^.

Genetic research indicates that the *Teshik Tash* individual from Central Asia belongs to the early Neanderthal haplogroup NA. At the present stage of research, the Teshik Tash Mousterian industry represents one of the main MP cultural facies in the western part of Central Asia (Krivoshapkin, 2012). Earlier, researchers associated the lithic assemblages from Denisova Cave (200–100 ka) with Neanderthals and defined as Levallois Mousterian^76^. However, now representatives of two ancient populations (Neanderthals and Denisovans) have been identified in Denisova Cave, and researchers claim it is not possible to separate lithic industries associated with Neanderthals or Denisovans due to their technological and typological homogeneity^73^.

**SUPPLEMENTARY FIGURES AND TABLES**


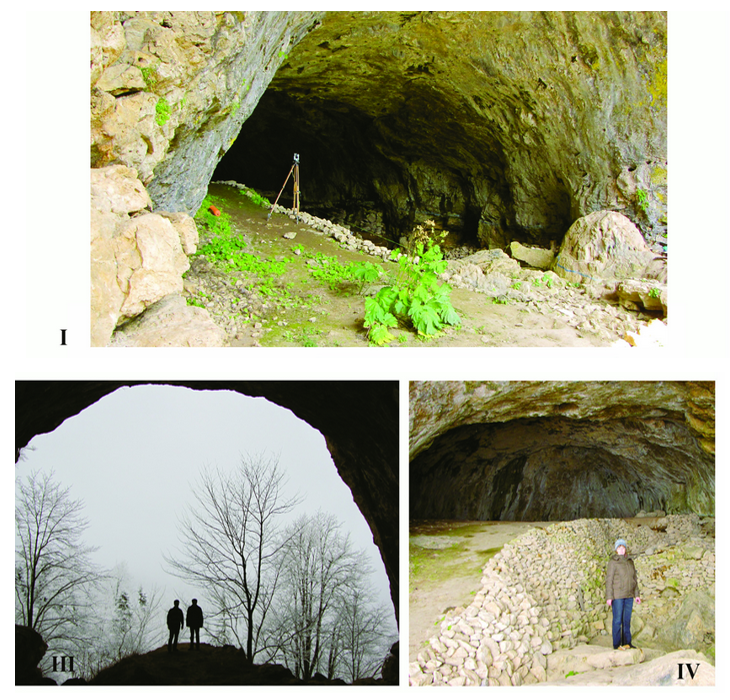


Supplementary Figure S1. I—Mezmaiskaya Cave. View from the southwest.

IIШ. View from Mezmaiskaya Cave in the winter time. View from the northeast.

IV. View on excavation in Mezmaiskaya Cave. View from the southeast.


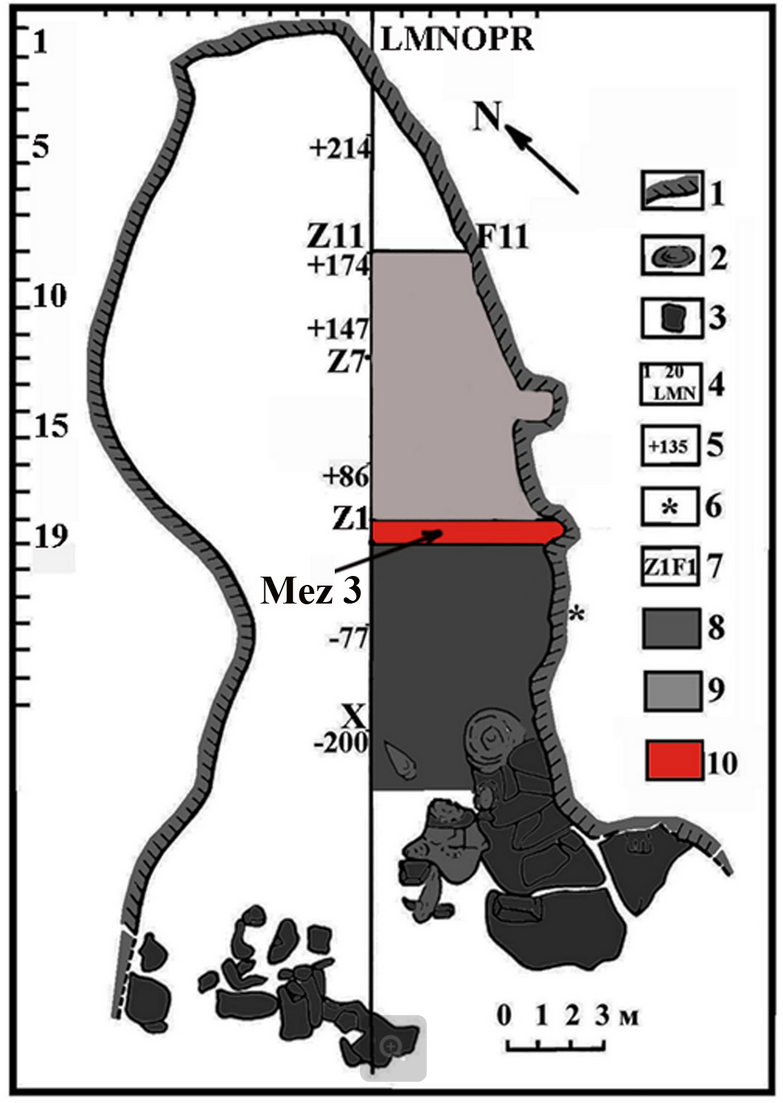


Supplementary Figure S2. Plan of Mezmaiskaya Cave. Legend: 1—bedrock, 2—flowstones, 3—limestone blocks, 4—grid nomenclature, 5—measurements of the modern surface along the cave axis, 6—datum, 7—indicators for sections, 8—excavation area of Middle Palaeolithic layers, 9—excavation area on which only post-Palaeolithic and Upper Palaeolithic layers are excavated, 10—excavation area of Middle Palaeolithic layer 3 shown in Supplementary Figure S4–I.


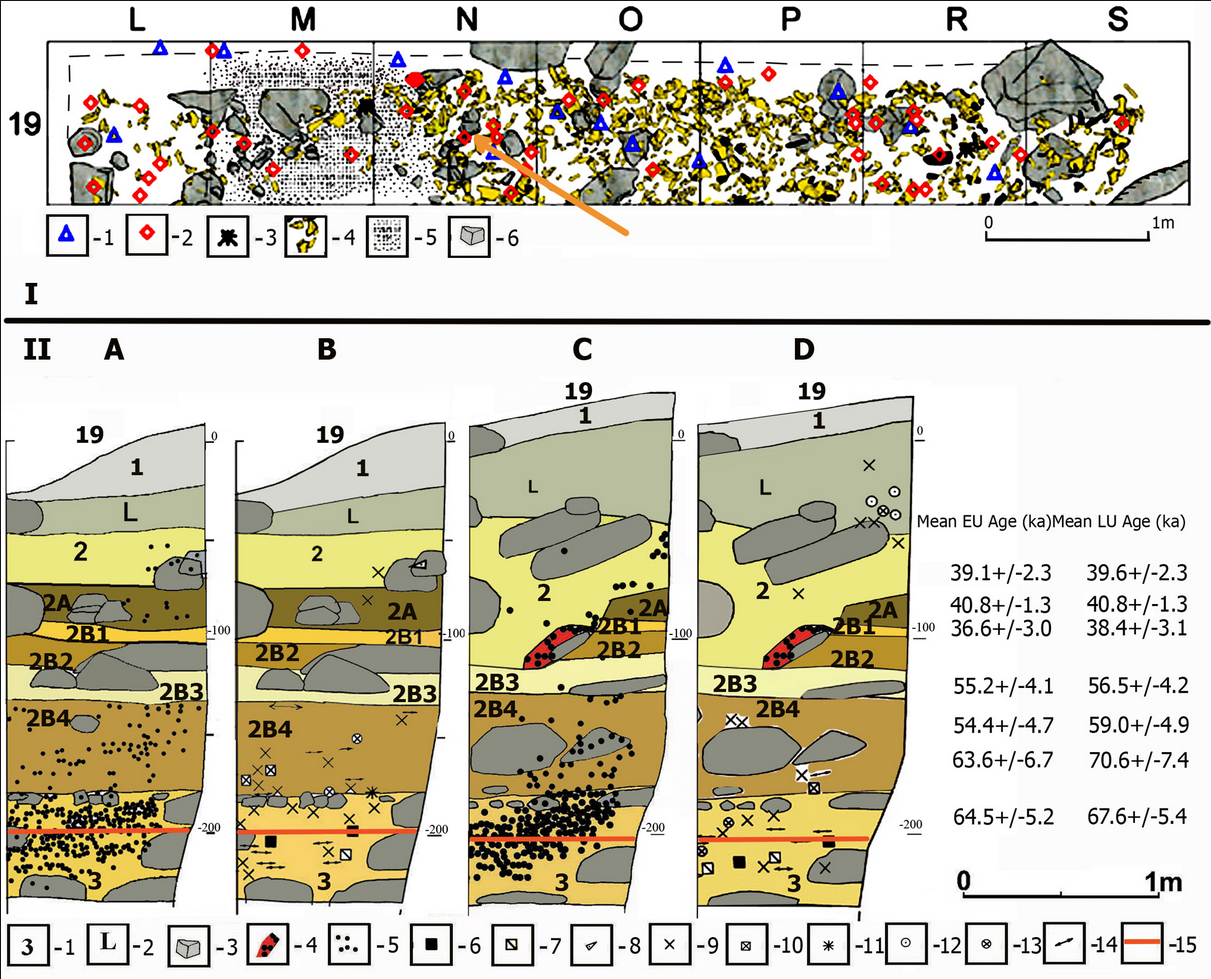


Supplementary Figure S3. I. Plan of the area of layer 3 along quadrant Line L-P–19 indicating the position of *Mezmaiskaya 3* (shown by arrow).

Legend: 1—tools, 2—flaking debris, 3—chips, 4—bones, 5—hearth, 6—limestone blocks.

II. Microprofiles running along the eastern (A, B) and western (C, D) parts of quadrant N-19 with mean ESR dates for Middle Palaeolithic deposits.

Legend: 1—layer designations, 2—eroded deposits, 3—stones, 4— stratigraphic position of *Mezmaiskaya 2*, 5—bones, 6—cores, 7—split nodules, 8—points, 9—flakes, 10—flake fragments, 11—chips, 12—bladelets, 13—bladelet fragments, 14—tools, 15—stratigraphic position of *Mezmaiskaya 3*.


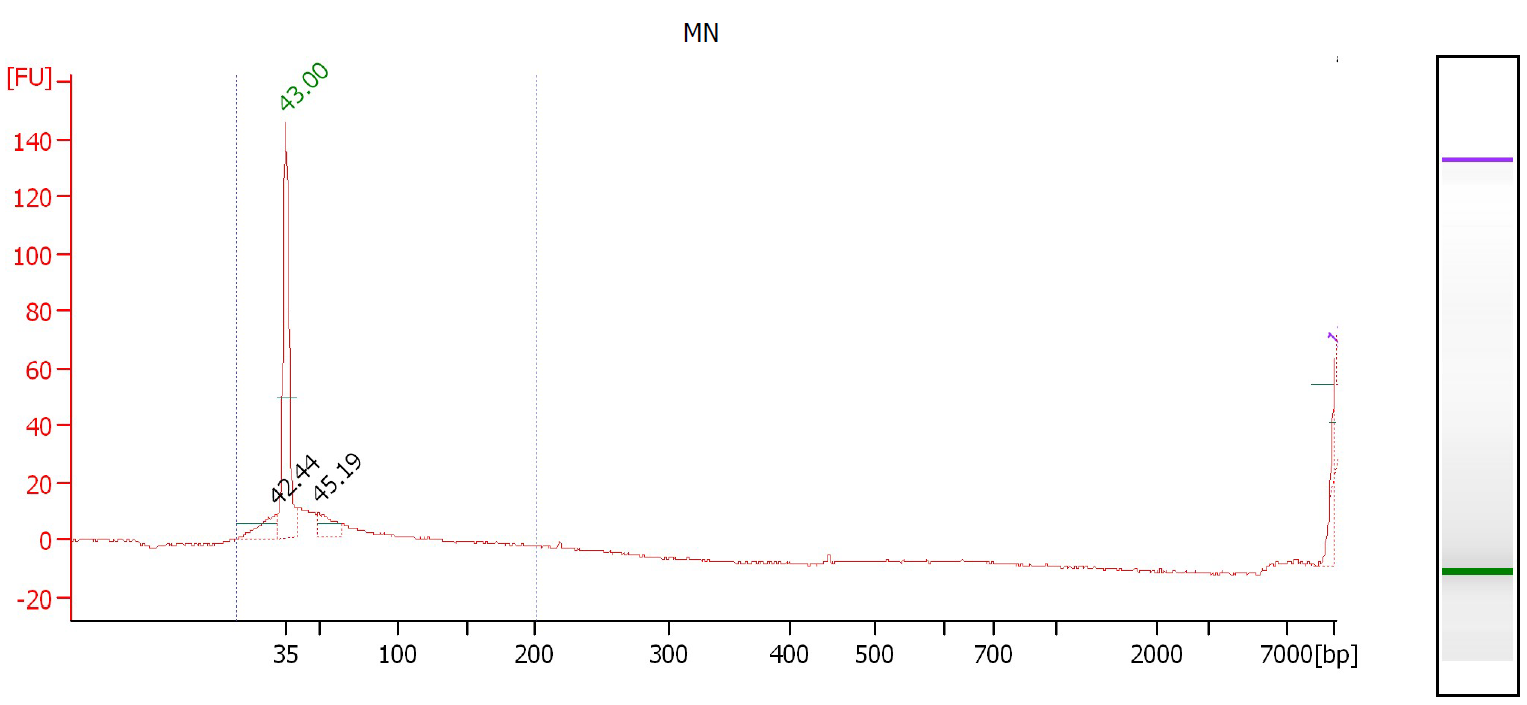

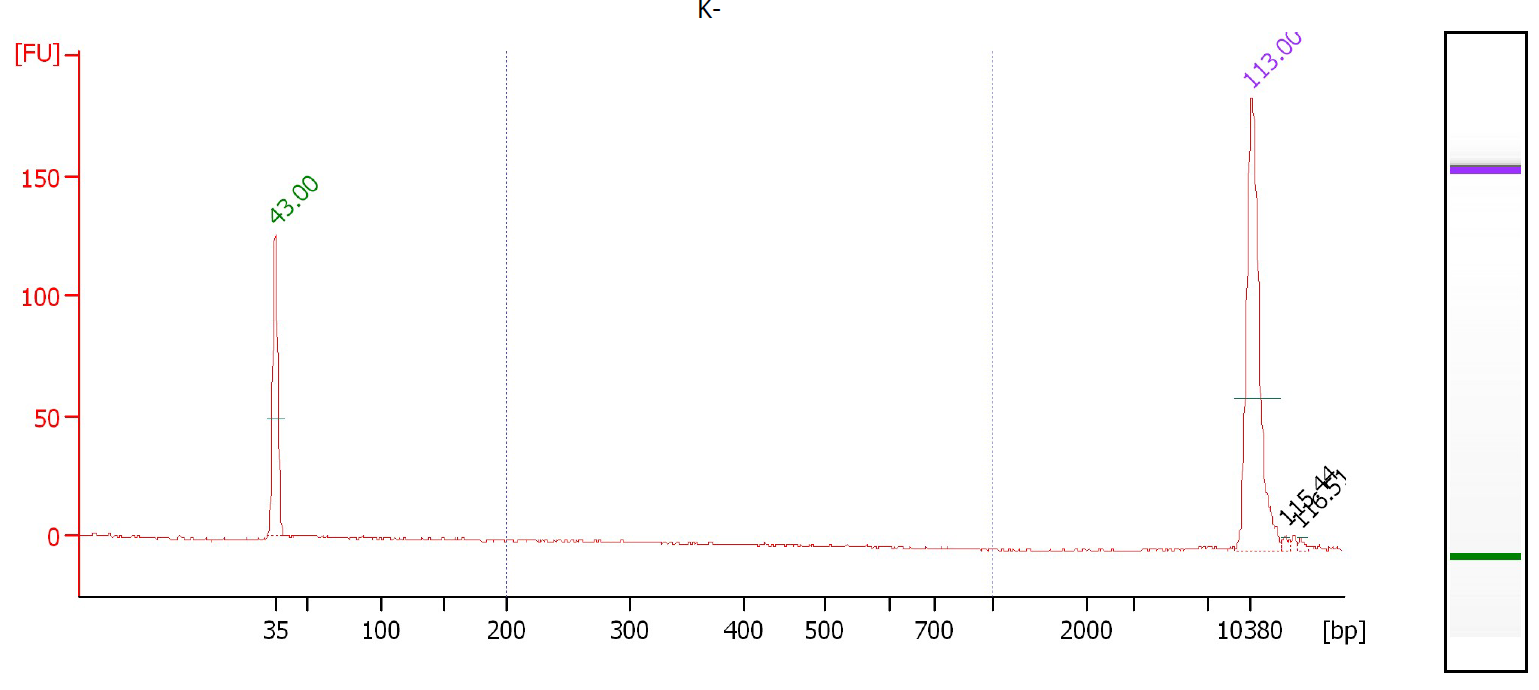


Supplementary Figure S4. Agilent Bioanalyzer 2100 analysis result of the *Mezmaiskaya 3* DNA (MN, left) and blank control (K-, right). A small peak corresponding to DNA fragments less than 50 bp was visualized in the *Mezmaiskaya 3* DNA sample.

A


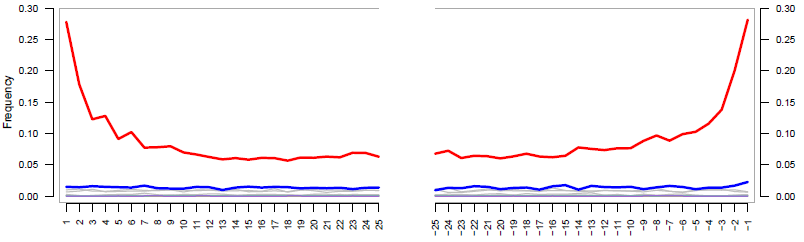


B


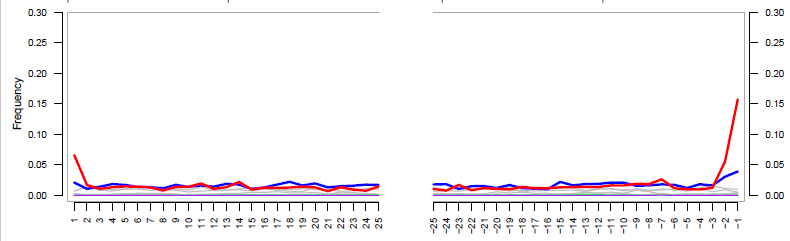


Supplementary Figure S5. Base substitutions in the sequenced DNA libraries of the *Mezmaiskaya 3* specimen are related to the postmortem degradation pattern of ancient DNA: A—untreated DNA; B—PreCR-treated DNA (New England Biolabs, USA). Ancient DNA-specific increases in C to T (red) and G to A (blue) changes towards the ends of the reads were observed, and PreCR treatment decreased the total C to T change rate. 35 bp read length cut-off was used for this analysis.


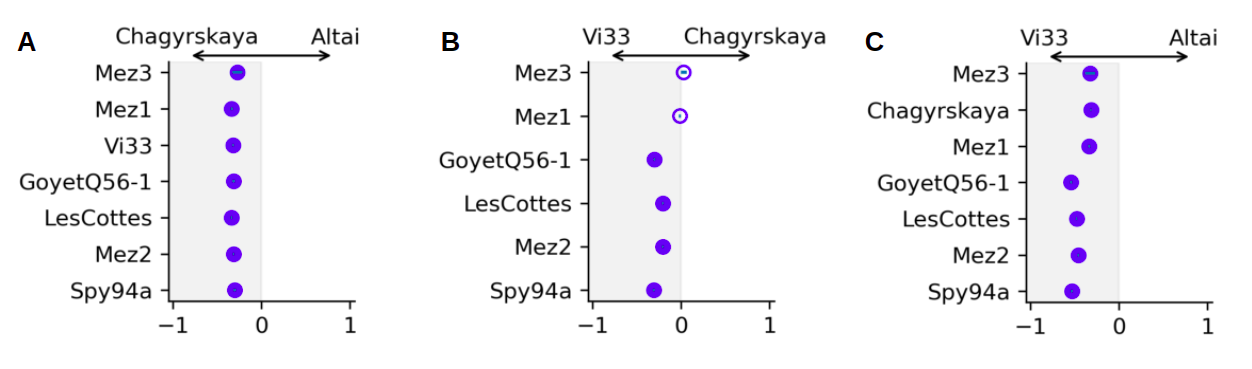
Supplementary Figure S6. Relationship of *Mezmaiskaya 3*, *Mezmaiskaya 1* and the late Neanderthals to the *Altai*, *Chagyrskaya 8* and *Vindija 33.19* Neanderthals computed as D(Altai, Chagyrskaya, Neanderthal, Chimpanzee) (A), D(Chagyrskaya, Vindija 33.19, Neanderthal, Chimpanzee) (B) and D(Altai, Vindija 33.19, Neanderthal, Chimpanzee) (C) for transversions in map35_100 regions called using putatively deaminated fragments only. *Mezmaiskaya 3* and all other Neanderthals in this analysis are significantly closer to *Vindija 33.19* (A) and *Chagyrskaya 8* (C) than to the *Altai* Neanderthal. When compared to *Chagyrskaya 8* and *Vindija 33.19* (B) all late Neanderthals are significantly closer to *Vindija 33.19*, while *Mezmaiskaya 3* and *Mezmaiskaya 1* D-statistics values cannot be significantly distinguished from zero (D±se = -0.03±0.03355, Z = 0.893 and D±se = -0.0111±0.01161, Z = -0.956 correspondingly). Filled dots denote statistical significance (Z < -2). Mez1, 2, 3 – *Mezmaiskaya 1, 2, 3* samples; Vi33 – *Vindija33.19*.


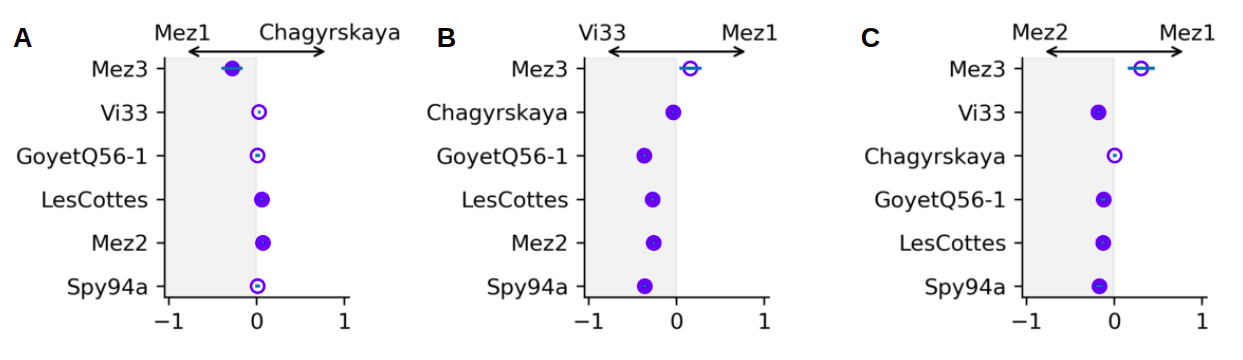


Supplementary Figure S7. Relationship of *Mezmaiskaya 3* and Neanderthals from distinct time periods (Middle Neanderthals: *Mezmaiskaya 1* and *Chagyrskaya* *8* and late Neanderthals: *Vindija33.19* and *Mezmaiskaya 2*) computed as D(Neanderthal1, Neanderthal2, Test, Chimpanzee) for transversions in map35_100 regions called using putatively deaminated fragments only. *Mezmaiskaya 3* demonstrates higher genetic affinity to *Mezmaiskaya 1* in comparison to *Chagyrskaya 8*.


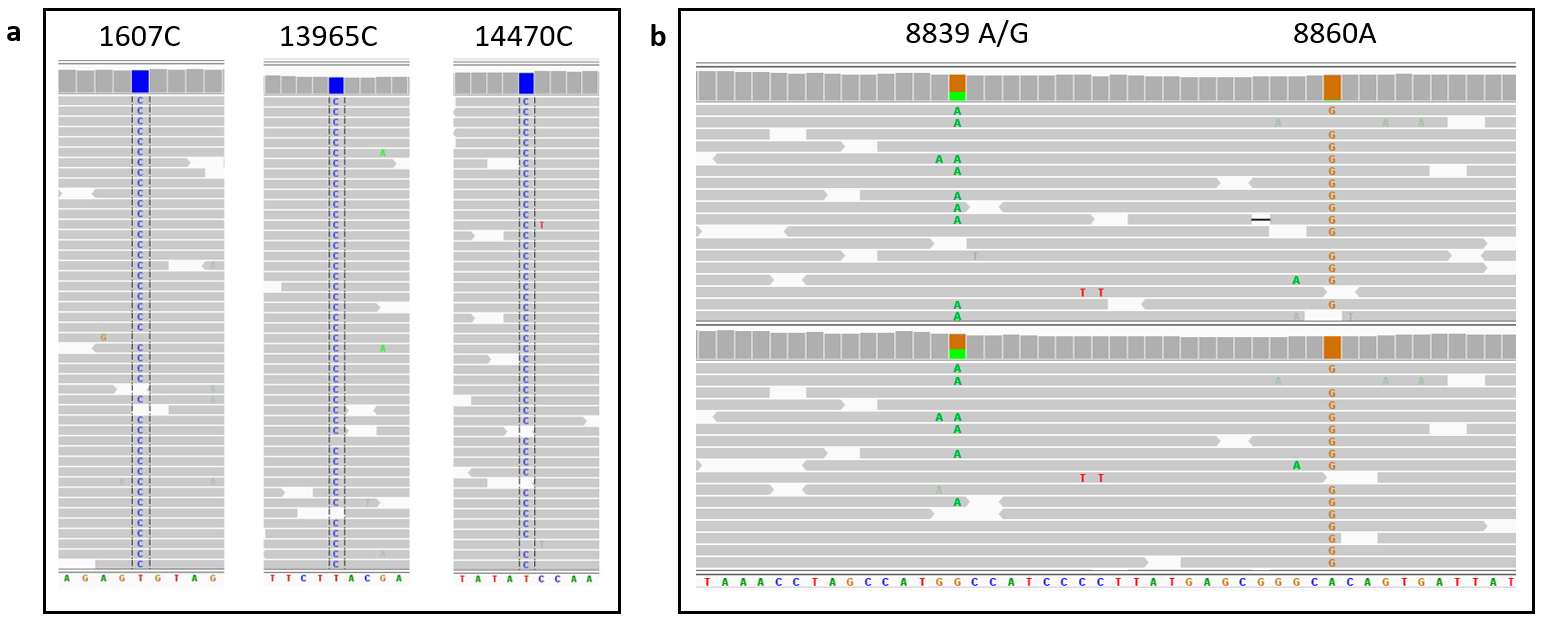


Supplementary Figure S8. Specific mitochondrial DNA variants for *Mezmaiskaya 3* visualized in The Integrative Genomics Viewer (IGV)^77^. The majority of sequences at mitochondrial DNA positions 1607, 13965, and 14470 carry allele C and have no signs of contamination. Single T allele at these three positions most likely represent the ancient postmortem C>T substitutions (**a**). Both A and G variants (**b**) are present at position 8839 and represent heteroplasmy. Reads with MQ>=30 are presented for DNA fragments longer than 25 (upper panel) and 35 (lower panel) nucleotides. 8860A variant is shared by all Neanderthals.


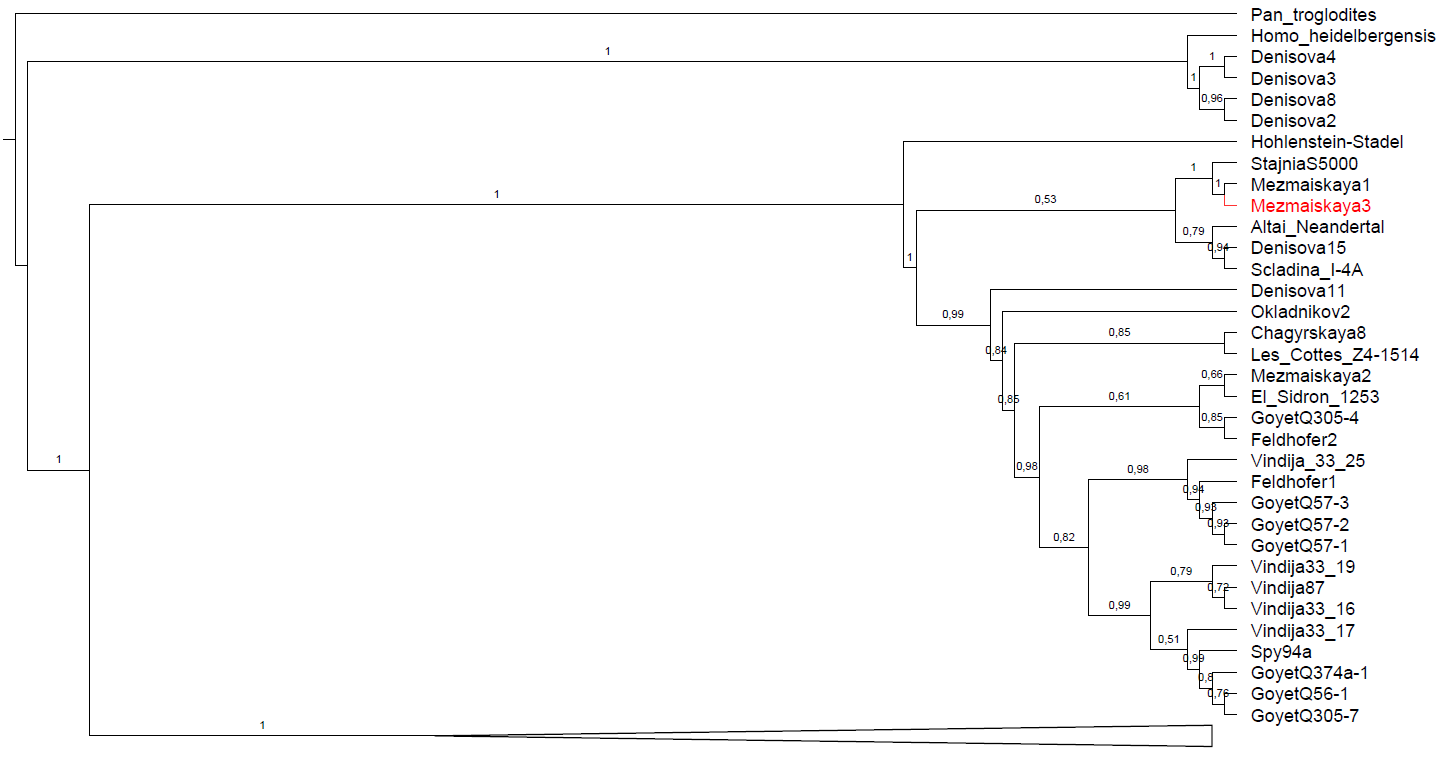


Supplementary Figure S9—Maximum parsimony tree relating the *Mezmaiskaya 3* mitochondrial genome to archaic and modern human mitochondrial genomes. Evolutionary analyses were conducted for coding mitochondrial region sequences in MEGA X^78^. Supports from 1000 bootstrap replicates are shown. The branches corresponding to modern human mtDNAs collapsed in the bottom.


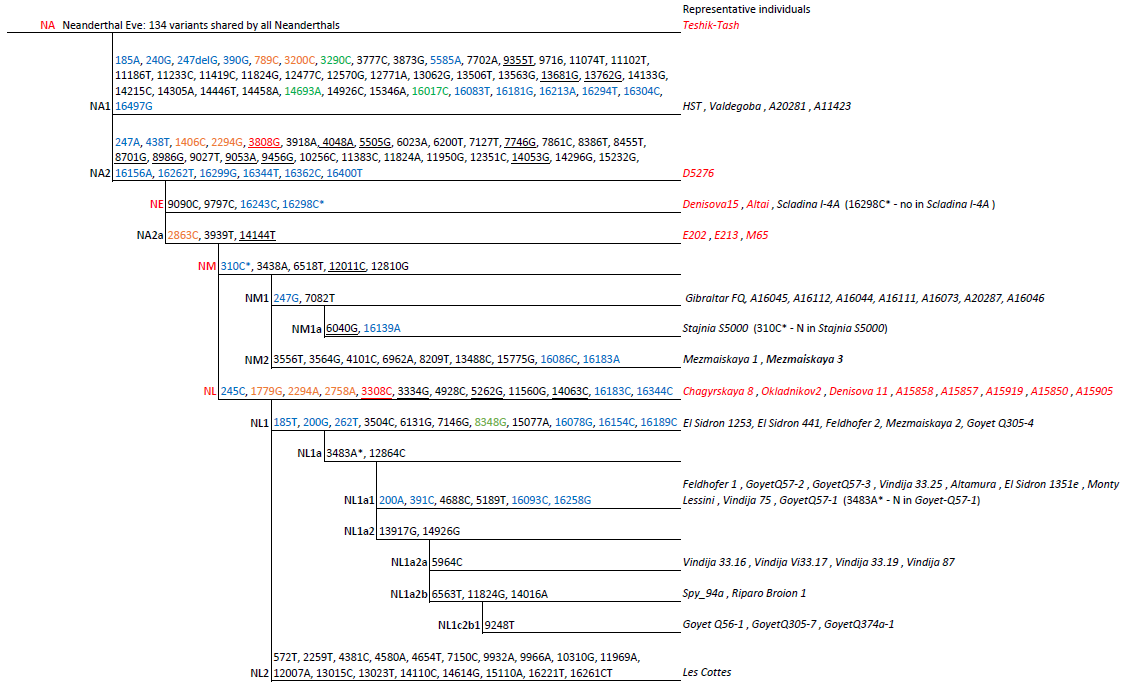


Supplementary Figure S10. Phylogenetic tree of Neanderthal mitochondrial DNA haplogroups. Nucleotide position numbers are consistent with both the rCRS and the RSRS reference sequences. Coding-region variations are shown in black with and underlined mutations with amino acid changes; intergenic and control-region mutations are shown in blue; tRNA mutations are shown in green; and rRNA mutations are shown in brown.

Representative Neanderthals from Asia are shown in red; individuals from Europe are shown in black.

HST—Hohlenstein-Stadel.

D5276—Denisova Cave, Altai, Russia (Vernot et al., 2021).

A20281, A11423—Galería de las Estatuas, Spain (Vernot et al., 2021).

E202, E213, M65—Denisova Cave, Altai, Russia (Zavala et al., 2021).

A16045, A16112, A16044, A16111, A16073, A20287, A16046—Galería de las Estatuas, Spain (Vernot et al., 2021).

A15858, A15857, A15919, A15850, A15905—Chagyrskaya Cave, Altai, Russia (Vernot et al., 2021).

А
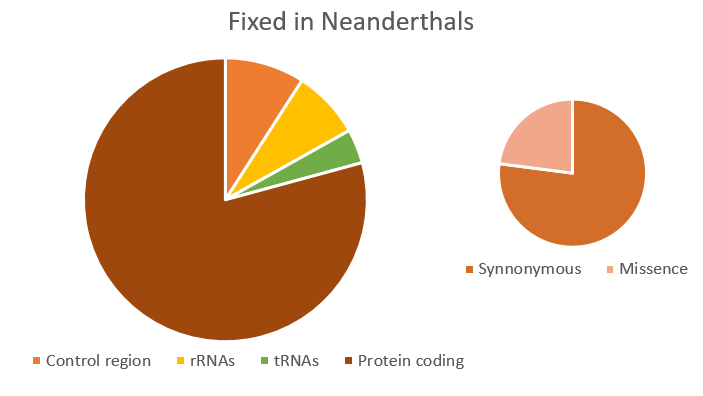
 B
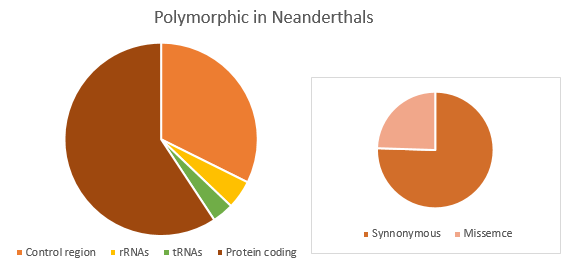


Supplementary Figure S11. Fixed (A) and polymorphic (B) mtDNA variants in Neanderthals.

A
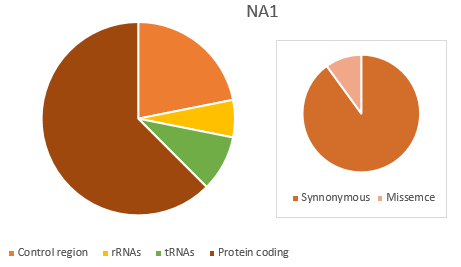
 B
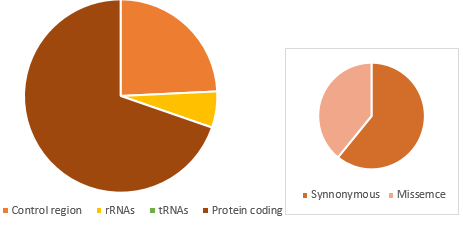


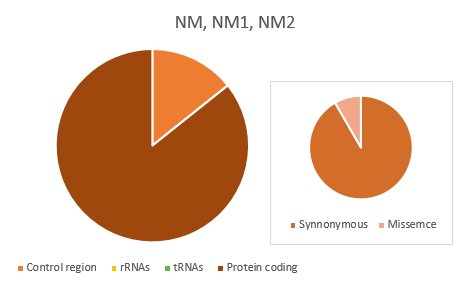


C

Supplementary Figure S12. NA1 (A), NA2 (B), MN (С) mtDNA haplogroup variants in Neanderthals.


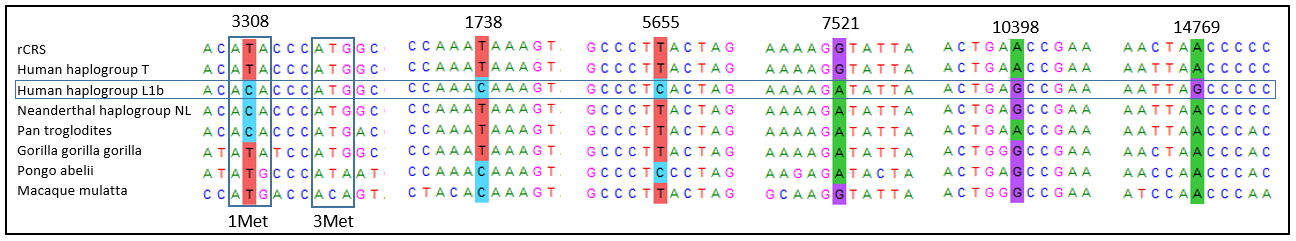


Supplementary Figure S13. mtDNA coding region variants linked to the T3308C mutation in modern human haplogroup L1b contain different alleles in primates. Neanderthal’s allele C have unique background among primates.

Supplementary Table S1. List of the performed sequencing runs.

| **Library ID** | **RunType*** | **Read length** | **No. of raw sequences** | **No. of raw base** | **Mean length of retained reads** |
| --- | --- | --- | --- | --- | --- |
| MN_norep | PE | 76+76 | 16 568 869 | 1 690 024 638 | 59 |
|  | PE | 76+76 | 98 917 199 | 15 035 414 248 | 59 |
|  | SR | 76 | 118 644 951 | 9 017 016 276 | 54 |
| MN_rep | PE | 51+51 | 28 372 410 | 4 312 606 320 | 53 |
|  | PE | 76+76 | 91 747 551 | 13 945 627 752 | 60 |
|  | SR | 76 | 129 392 803 | 9 833 853 028 | 49 |
| **Total** |  |  | **483 643 783** | **53 834 542 262** |  |

# MN_norep—library prepared from untreated DNA; MN_rep—library prepared from PreCE MIX-treated DNA.

* PE—paired-end; SR—single-read.

Supplementary Table S2. Sequencing summary statistics of sequences generated from the *Mezmayskaya 3* Neanderthal individual.

| **Library ID#** | **Read length** | **Total reads** | **GRCh37 mapped reads (MQ>=25)** | **% GRCh37 mapped reads (MQ>=25)** | **Covered (MQ>=25)** | **Covered (MQ>=25, map35_100*)** | **Deaminated GRCh37 mapped reads (MQ>=25)** | **% Deaminated GRCh37 mapped reads (MQ>=25)** | **Deaminated covered (MQ>=25)** | **Deaminated covered (MQ>=25, map35_100)** |
| --- | --- | --- | --- | --- | --- | --- | --- | --- | --- | --- |
| MN_norep | >= 25 | 145 944 312 | 26 957 677 | 18,47% | 558 410 025 | 521 756 468 | 7 065 182 | 4,84% | 173 477 272 | 163 516 721 |
| MN_rep | >= 25 | 158 137 898 | 14 745 563 | 9,32% | 174 025 965 | 162 126 021 | 1 246 641 | 0,79% | 16 497 153 | 15 607 555 |
| Total | >= 25 | 304 082 210 | 41 703 240 | 13,71% | 684 818 231 | 639 485 425 | 8 311 823 | 2,73% | 188 460 374 | 177 698 581 |
| MN_norep | >= 35 | 73 347 171 | 7 673 748 | 10,46% | 232 444 797 | 213 397 054 | 2 420 707 | 3,30% | 76 266 056 | 70 385 638 |
| MN_rep | >= 35 | 97 629 840 | 3 142 121 | 3,22% | 54 223 864 | 49 133 346 | 328 225 | 0,34% | 5 674 381 | 5 209 923 |
| Total | >= 35 | 170 977 011 | 10 815 869 | 6,33% | 280 818 947 | 257 315 467 | 2 748 932 | 1,61% | 81 715 376 | 75 401 036 |

# MN_norep—library prepared from untreated DNA; MN_rep—library prepared from PreCE MIX-treated DNA.

*** map35_100 -** highly mappable regions of human genome (Prüfer et al., 2014)

### Supplementary Table S3. Expected ratio of X chromosomes to autosome fragments for *Mezmaiskaya 3* individuals. Only sequences with mapping quality >=25 were used for analysis.

| **DNA fragments** | | **Mean autosome coverage** | **Mean Х chromosome coverage** | **Ratio of  X chromosome coverage/autosome coverage** |
| --- | --- | --- | --- | --- |
| >=25 nt | All sequences | 1,32E-02 | 4,24E-01 | 0,93 |
|  | Deaminated sequences | 4,52E-03 | 4,17E-03 | 0,92 |
| >=35 nt | All sequences | 3,42E-03 | 3,51E-03 | 1,03 |
|  | Deaminated sequences | 1,45E-03 | 1,47E-03 | 1,01 |

Supplementary Table S4. Point estimates for fractions of sample-specific derived alleles that *Mezmaiskaya 3* share with high-coverage Neanderthals, Denisovan, and present-day human genome.
For each high-coverage genome, sample-specific autosomal SNPs (i.e., not found in other four samples) in highly mappable regions were used. Transitions were excluded. Ancestral state was estimated from EPO 6-way primate alignment requiring the same allele for *Pan troglodytes*, *Pongo abelii*, *Macaca mulatta,* and *Gorilla gorilla*. Only putatively deaminated fragments longer than 35 bp with high mapping quality (MAPQ ≥ 25) were considered. HGDP00982 is a present-day African (Mbuti).

| **Branch** | **Shared derived alleles (%)** | **95% binomial confidence interval (%)** | **Matched/total SNPs** |
| --- | --- | --- | --- |
| HGDP00982, Denisova 3, Altai, Chagyrskaya 8, Vindija 33.19 | 99.82 | 99.77–99.86 | 30346/30400 |
| Denisova 3, Altai, Chagyrskaya 8, Vindija 33.19 | 94.29 | 92.61–95.61 | 875/928 |
| Altai, Chagyrskaya 8, Vindija 33.19 | 89.70 | 88.02–91.17 | 1289/1437 |
| Chagyrskaya 8, Vindija 33.19 | 48.55 | 43.05–54.09 | 151/311 |
| Chagyrskaya 8 | 17.18 | 14.19–20.64 | 90/524 |
| Vindija 33.19 | 13.26 | 10.77–16.21 | 79/596 |
| Altai | 7.56 | 5.68–10.00 | 44/582 |
| HGDP00982 | 0.37 | 0.25–0.57 | 22/5867 |
| Denisova 3 | 0.34 | 0.19–0.61 | 11/3205 |

Supplementary Table S5. Results of the alignment of trimmed reads of length >=25 nt on the rCRS (NC_012920.1) and Neanderthal (NC_011137.1) mitochondrial DNA reference sequences.

| **Library ID#** | **Total reads** | **Total rCRS/Neanderthal mtDNA mapped reads** | **Mean length of mapped reads rCRS/Neanderthal mtDNA, bp** | **Duplicates rCRS/Neanderthal mtDNA** | **rCRS/Neanderthal mtDNA coverage, MAPQ>=25** |
| --- | --- | --- | --- | --- | --- |
| MN_norep | 145 944 312 | 681 347/682 627 | 26,18/26,23 | 96,54%/96,48% | 58,71/60,10 |
| MN_rep | 158 137 898 | 410 090/409 597 | 26,37/26,38 | 96,86%/96,84% | 30,97/31,15 |
| TOTAL | 304 082 210 | 1 091 437/1 092 224 | 26,25/26,29 | 96,66%/96,62% | 89,68/91,25 |

# MN_norep—library prepared from untreated DNA; MN_rep—library prepared from PreCR MIX-treated DNA.

Supplementary Table S6. Total set of Neanderthal mitochondrial DNA variants (see separate file ST_S6.xls). Sheet 1 (Neand<0,1)—common Neanderthals variants, Sheet 2 (polym_2Neand)—polymorphic variants shared by at least two Neanderthals.

Supplementary Table S7. Present-day human contamination based on Neanderthal- or modern-human-specific differences in mitochondrial genome sequences.

| **Library ID^#^** | **Reference** | **Neanderthal variant count 25 bp/35 bp** | **Modern human variant count 25pb/35 bp** | **Modern human sequences contamination 25/35 bp (%)** |
| --- | --- | --- | --- | --- |
| MN_norep | NC_012920.1 | 1453/1140 | 155/106 | 5,3/4,4 |
|  | NC_011137.1 | 3772/2925 | 43/41 | 1,2/1,6 |
| MN_norep (deaminated fragments) | NC_012920.1 | 560/451 | 57/38 | 6,1/4,9 |
|  | NC_011137.1 | 1636/1291 | 26/6 | 1,6/1,3 |
| MN_rep | NC_012920.1 | 1877/1230 | 49/32 | 2,7/2,8 |
|  | NC_011137.1 | 2042/1434 | 19/12 | 0,9/0,9 |
| MN_rep (deaminated fragments) | NC_012920.1 | 453/167 | 0/0 | - |
|  | NC_011137.1 | 178/113 | 0/0 | - |
| Total (all fragments) | NC_012920.1 | 4787/3410 | 201/134 | 3,9/3,7 |
|  | NC_011137.1 | 5821/4360 | 45/47 | 0,8/1,18 |
| Total (deaminated fragments) | NC_012920.1 | 2174/1588 | 57/38 | 3,1/2,9 |
|  | NC_011137.1 | 1817/1407 | 28/16 | 1,5/1,15 |

# MN_norep—library prepared from untreated DNA; MN_rep—library prepared from PreCR MIX-treated DNA.

Supplementary Table S8. Specific mtDNA variants of *Mezmaiskaya 3.*

| rCRS position | Human Reference allele | *Mezmaiskaya 3* variants | GnomAD allele frequencies | philoP score (interpretation)^#^ |
| --- | --- | --- | --- | --- |
| 1607 | T | C | 0.0003722 | -1,817 (Fast-evolving) |
| 8839 | G | A, G* | 0.001028 (G) | 6,346 (Conserved) |
| 16965 | T | C | 0.005086 | -7,544 (Fast-evolving) |
| 14470 | T | C | 0.01564 | -4,557 (Fast-evolving) |

*—Heteroplasmy.

#—Basewise conservation of 100 vertebrates by PhyloP (phyloP100way)^79^.

Supplementary Table S9. Allele count for the 8839 position of the human reference genome in the *Mezmaiskaya 3* specimen.

| **Library^#^** | **Reference** | **A**  **25 bp/35 bp** | **G (ref)**  **25pb/35 bp** | **G allele frequency 25/35 bp (%)** |
| --- | --- | --- | --- | --- |
| MN_norep | NC_012920.1 | 16/12 | 32/23 | 66,67/65,71 |
|  | NC_011137.1 | 16/12 | 32/23 | 66,67/65,71 |
| MN_norep (deaminated fragments) | NC_012920.1 | 6/4 | 11/6 | 64,71/60,00 |
|  | NC_011137.1 | 6/4 | 11/6 | 64,71/60,00 |
| MN_rep | NC_012920.1 | 11/7 | 25/15 | 69,44/68,18 |
|  | NC_011137.1 | 8/7 | 25/15 | 75,76/68,18 |
| MN_rep (deaminated fragments) | NC_012920.1 | 3/0 | 1/0 | 25/0 |
|  | NC_011137.1 | 0/0 | 2/0 | - |
| Total (all fragments) | NC_012920.1 | 27/19 | 57/38 | 67,86/66,67 |
|  | NC_011137.1 | 29/19 | 57/38 | 66,28/66,67 |
| Total (deaminated fragments) | NC_012920.1 | 12/10 | 12/6 | 50,00/37,50 |
|  | NC_011137.1 | 6/4 | 12/6 | 66,67/60,00 |

# MN_norep—library prepared from untreated DNA; MN_rep—library prepared from PreCR MIX-treated DNA.

Supplementary Table S10. Number of pairwise nucleotide differences per sequence between the complete mtDNA sequences of humans, Neanderthals, Denisovans, and the chimpanzee. All ambiguous positions were removed for each sequence pair (pairwise deletion option). There were a total of 16596 positions in the final dataset. Evolutionary analyses were conducted in MEGA X^80^ (see separate file ST_S8.xls).

Supplementary Table S11. Radiocarbon dates used in the Bayesian phylogenetic analyses.

| **Individual** | **Date (CI)** | **Gene bank ID** | **Ref** |
| --- | --- | --- | --- |
| **Modern human** | | | |
| Tianyuan | 39,008 (37,761–40,254) | KC417443 | Fu, Meyer et al, 2013 |
| Kostenki 14 | 37,473 (36,262–38,684) | FN600416 | Krause et al, 2010 |
| Dolní Věstonice 13 | 31,071 (30,884–31,249) | KC521459 | Fu, Mittnik et al, 2013; Fewlass et al, 2019 |
| Dolní Věstonice 14 | 30,934 (30,741–31,120) | KC521458 | Fu, Mittnik et al, 2013; Fewlass et al, 2019 |
| Oberkassel 998 | 14,077 (13,755–14,105) | KC521457 | Fu, Mittnik et al, 2013 |
| Boshan 11 | 8,234 (8,152–8,316) | KC521454 | Fu, Mittnik et al, 2013 |
| Loschbour | 8,054 (7,948–8,160) | KC521455 | Fu, Mittnik et al, 2013 |
| Iceman | 5,300 (5,275–5,325) | EU810403 | Ermini et al, 2008 |
| Saqqaq Eskimo | 4,504 (4,423–4,585) | EU725621 | Gilbert et al, 2008 |
| Ust‘-Ishim | 45,045 (43,212–46,878) | - | Fu et al, 2014 |
| **Neanderthals** | | | |
| Goyet Q305–4 | 44,236 (43,386–45,085) | KX198087 | Rougier et al, 2016 |
| Mezmaiskaya 2 | 43,834 (42,038–45,630) | MG025537 | Hajdinjak et al, 2018; Pinhasi et al, 2011 |
| Feldhofer 1 | 43,707 (42,670–44,744) | FM865407 | Briggs et al, 2009; Schmitz et al, 2002 |
| Feldhofer_2 | 43,268 (42193–44342) | FM865408 | Briggs et al, 2009; Schmitz et al, 2002 |
| Vindija 33.16 | 43,707 (39,234–48,179) | AM948965 | Green et al, 2008; Serre et al, 2004 |
| Les Cottés Z4–1514 | 43,230 (42,720–43,740) | MG025536 | Hajdinjak et al, 2018 |
| Goyet Q56–1 | 42,515 (42,03–42,967) | KX198084 | Rougier et al, 2016 |
| GoyetQ57–1 | 44696 (43834–45558) | KX198082 | Rougier et al, 2016 |
| Goyet Q57–3 | 42,407 (41,964–42,867) | KX198083 | Rougier et al, 2016 |
| Goyet Q57–2 | 41,185 (40,595–41,775) | KX198088 | Rougier et al, 2016 |
| Spy 94a | 40,463 (39,234–48,179) | MG025538 | Hajdinjak et al, 2018; Semal et al, 2009 |

Supplementary Table S12. Estimated molecular age and divergence time specimens obtained from four independent MCMC runs of 75 000 000 iterations each.

| **Individual** | **Mean** | **95% HPD interval** | **ESS** |
| --- | --- | --- | --- |
| Denisova 2 | 221024.703 | 157643.822–90800.537 | 1080.7 |
| Denisova 8 | 185836.7105 | 112537.533–267353.128 | 1217.2 |
| Denisova 3 | 85296.3532 | 60721.8084–99999.9374 | 663.8 |
| Denisova 4 | 90562.6301 | 59768.186–116068.9072 | 634.8 |
| Hohlenstein-Stadel | 114423.998 | 60054.6759–169835.4367 | 4971.4 |
| Altai Neanderthal | 132410.608 | 93866.8725–169830.3197 | 1244.6 |
| Denisova 15 | 128599.458 | 89614.5225–166892.6663 | 1294.4 |
| Scladina I–4a | 124873.116 | 85822.1372–163607.3502 | 1329 |
| Stajnia S5000 | 125478.731 | 90233.9639–161746.2711 | 1236.4 |
| Mezmayskaya 1 | 107606.862 | 70379.872–144522.9535 | 1165.4 |
| **Mezmayskaya 3** | **96795.012** | **58877.1874–133999.4879** | **1299.7** |
| Denisova 11 | 102068.338 | 76517.8853–129004.5033 | 1644.2 |
| Okladnikov 2 | 94767.8196 | 67667.5113–123031.8551 | 2068.5 |
| Chagyrskaya 8 | 84650.5906 | 56934.7945–113539.6382 | 2558 |
| El_Sidron_1253 | 58270.8879 | 40734.7967–77705.2848 | 8925.4 |
| Vindija33_17 | 51746.6638 | 42948.8769–61214.3837 | 7667 |
| Vindija33_19 | 44791.442 | 34806.0173–53863.7593 | 8315.4 |
| Vindija87 | 44765.379 | 34635.2386–53761.7868 | 8279.1 |
| Vindija_33_25 | 44152.0202 | 33793.9508–54803.5489 | 10231.2 |
| GoyetQ305–7 | 40795.1327 | 32657.3088–47064.2689 | 25464.4 |
| GoyetQ374a-1 | 40817.5387 | 32621.0773–47089.6532 | 24857.3 |
| Neanderthals and modern human TMRCA | 3.8626E5 | 3.3695E5–4.3746E5 | 5095.7 |
| Neanderthals TMRCA | 2.5755E5 | 2.1524E5–3.0133E5 | 2424.7 |
| Haplogroup NM TMRCA | 1.4394E5 | 1.1147E5–1.768E5 | 1014 |
| Haplogroup NL TMRCA | 1.1329E5 | 91642.2684–1.373E5 | 1330.9 |

Supplementary Table S13.—Neanderthal mtDNA haplogroup markers (see separate file ST_S13.xls).

Supplementary Table S14. Mitochondrial DNA haplogroup estimates for Neanderthal genomes from northern Spain and Altai.

| **Neanderthal individual** | **Reference** | **Geographic source** | **Haplogroup** |
| --- | --- | --- | --- |
| A16045 | Vernot et al, 2021 | Galería de las Estatuas, Spain | NM1 |
| D5276 | Vernot et al, 2021 | Denisova Cave, Altai, Russia | NA2 |
| A20281 | Vernot et al, 2021 | Galería de las Estatuas, Spain | NA1 |
| A16112 | Vernot et al, 2021 | Galería de las Estatuas, Spain | NM1 |
| A15858 | Vernot et al, 2021 | Chagyrskaya Cave, Altai, Russia | NL |
| A16044 | Vernot et al, 2021 | Galería de las Estatuas, Spain | NM1 |
| A15857 | Vernot et al, 2021 | Chagyrskaya Cave, Altai, Russia | NL |
| A15919 | Vernot et al, 2021 | Chagyrskaya Cave, Altai, Russia | NL |
| A16111 | Vernot et al, 2021 | Galería de las Estatuas, Spain | NM1 |
| A16073 | Vernot et al, 2021 | Galería de las Estatuas, Spain | NM1 |
| A15850 | Vernot et al, 2021 | Chagyrskaya Cave, Altai, Russia | NL |
| A20287 | Vernot et al, 2021 | Galería de las Estatuas, Spain | NM1 |
| A11423 | Vernot et al, 2021 | Galería de las Estatuas, Spain | NA1 |
| A16046 | Vernot et al, 2021 | Galería de las Estatuas, Spain | NM1 |
| A15905 | Vernot et al, 2021 | Chagyrskaya Cave, Altai, Russia | NL |
| E202 | Zavala et al, 2021 | Denisova Cave, Altai, Russia | NA2a |
| E213 | Zavala et al, 2021 | Denisova Cave, Altai, Russia | NA2a |
| M65 | Zavala et al, 2021 | Denisova Cave, Altai, Russia | NA2a |

Supplementary Table S15. Mitochondrial DNA haplogroup estimates for low-coverage Neanderthal genomes and individuals with only control region variants.

| **Neanderthal individual** | **Reference or GenBank ID** | **mtDNA diagnostic markers (haplogroup)** | **Haplogroup** |
| --- | --- | --- | --- |
| Riparo Biron, Italy | ^46^ | Set of NL1a2b markers | NL1a2b |
| Forbes Quarry, Gibraltar | ENA PRJEB31410 | 310C (NM), 3438A (NM), 6518T (NM),12011C (NM), 12810G (NM), 247G (NM1), 5821A, 7082T (NM1) | NM1 |
| Altamura (Italy) | KJ888153.1 | 16258G (NL1a1) | NL1a1 |
| El Sidron 441, Spain | DQ859014.2 | 16154C (NL1), 16183C (NL), 16189C, 16258G (NL1) | NL1 |
| El Sidron 1351e, Spain | FM866397.1 | 16154C (NL1), 16183C (NL), 16189C (NL1), 16258G (NL1a1) | NL1a1 |
| Montу Lessini, Italy | DQ836132.1 | 16182C, 16183C (NL), 16189C (NL1), 16258G (NL1a1) | NL1a1 |
| Vindija 75 | AF282971.1 | 16154C (NL1), 16183C (NL), 16189C (NL1), 16258G (NL1a1) | NL1a1 |
| Valdegoba, Spain | JQ670672.1 | 16086C (NM2), 16156A (NA2), 16183C (NL), 16242T (NA1*), 16258C, 16294T (NA1*), 16304C (NA1*) | NA1* |
| Teshik Tash | EU078679.1 | 16156A (NA2), 16183del (NA), 16242T (NA1*), 16423C, 16266T, 16274A | NA (unknown clade?) |

* 16242T, 16294T, and 16304C are present in several low-quality and incomplete mtDNA sequences but could be related to the new NA1a haplogroup.

Supplementary Table S16. tRNA variants found in different Neanderthal haplogroups and their pathogenicity predictions.

| **rCRS position** | **Human Reference allele** | **Neanderthal allele** | **Locus** | **Haplogroup** | **HmtVar Pathogenicity Prediction** **(disease score)** | **MitoTip Ranking (Score)** | **Comments** |
| --- | --- | --- | --- | --- | --- | --- | --- |
| 3290 | T | C | MT-TL1 | NA1 | Likely polymorphic (0.01) | 1.5331 |  |
| 5821 | G | A | MT-TC | NA (common Neanderthal variant) | Polymorphic (0.03) | Likely benign (7.6717) |  |
| 5840 | C | T | MT-TY | NA (common Neanderthal variant) | Likely Polymorphic (0.01) | Likely benign (5.0089) |  |
| 8288 | TA | delTA | MT-TK | different haplogroups | - | - | Not present in HmtVar and MitoTip |
| 8348 | A | G | MT-TK | NL1 | Pathogenic (0.4) | Possibly benign (10.3301) | Cardiomyopathy  PMID: **11446509** |
| 12189 | T | C | MT-TH | NA (common Neanderthal variant) | Likely polymorphic (< 0.35) | Likely benign (2.54675) |  |
| 14693 | G | A | MT-TE | NA1 | Likely polymorphic (0.3) | Possibly benign (11.4662) |  |
| 16017 | T | C | MT-TP | NA1 | Likely polymorphic (< 0.35) | Possibly benign (10.0488) |  |

Supplementary Table S17. Diversity of mitochondrial DNA variants in different Neanderthal haplogroups.

|  | Fixed in Neanderthals* | At least two individuals | NA1 | NA2 | NE | Na2a | NM root | NM, NM1, NM2 | NL | NL1 |
| --- | --- | --- | --- | --- | --- | --- | --- | --- | --- | --- |
| Total | 77 | 168 | 41 | 32 | 4 | 3 | 5 | 16 | 13 | 11 |
| Neanderthals only | 4 | 16 | 5 | 1 | 0 | 2 | 0 | 1 | 1 | 0 |
| Control region, non-coding intragenic region | 7 | 55 | 10 | 7 | 2 | 0 | 1 | 4 | 4 | 6 |
| Ribosomal RNAs | 6 | 8 | 2 | 2 | 0 | 1 | 0 | 0 | 3 |  |
| tRNAs | 3 | 5 | 3 | 0 | 0 |  | 0 | 0 |  | 1 |
| Synonymous/missense | 47/14 | 75/24 | 23/3 | 14/9 |  | 1/1 | 3/1 | 11/1 | 2/3 | 2/2 |
| LoF |  | 1 (stop) |  |  |  |  |  |  | 1 (stop) |  |
| *ND1* | 3/0^#^ | 10/5 | 2/0 | 1/2 |  | 1/0 | 1/0 | 4/0 | 0/2 | 1/0 |
| *ND2* | 5/0 | 6/2 |  | 0/1 |  |  |  |  | 1/1 |  |
| *CO1* | 4/0 | 12/1 |  | 3/0 |  |  | 1/0 | 3/0 |  | 1/1 |
| *CO2* | 2/2 | 3/1 | 1/0 | 1/1 |  |  |  | 1/0 |  |  |
| *ATP8* | 2/1 | 2/0 |  | 2/0 |  |  |  |  |  |  |
| *ATP6* | 2/1 | 2/4 |  | 1/3 | 1/0 |  |  |  |  |  |
| *CO3* | 3/1 | 4/2 | 1/1 | 0/1 | 1/0 |  |  |  |  |  |
| *ND3* | 2/1 | 1/1 |  | 1/0 |  |  |  |  |  |  |
| *ND4L* | 3/1 | 2/0 |  |  |  |  |  |  |  |  |
| *ND4* | 4/0 | 10/1 | 6/0 | 3/0 |  |  | 0/1 | 0/1 | 1/0 |  |
| *ND5* | 8/5 | 14/6 | 7/2 | 1/1 |  | 0/1 | 1/0 | 2/0 | 0/1 |  |
| *ND6* | 1/0 | 5/0 | 4/0 | 1/0 |  |  |  |  |  |  |
| *CYTB* | 8/2 | 4/2 | 2/0 | 1/0 |  |  |  | 1/0 |  | 0/1 |

* Fixed in Neanderthals variants with MAF <0,1 in present-day humans are presented.

^#^ Synonymous/nonsynonymous variants are indicated for each gene**.**

**SUPPLEMENTARY REFERENCES**

1. Golovanova, L. V. Les hommes de Néandertal du Caucase du Nord: Entre l’Ouest et l’Est. *Anthropol.* **119**, 254–301 (2015).

2. Golovanova, L. V., Doronichev, V. B., Nedomolkin, A. G. & Doronicheva, E. V. Study of Upper Paleolithic sites in the North Caucasus. *Archaeol. Discov.* **2016**, 207–210 (2018).

3. Golovanova, L. V & Doronichev, V. B. *Environment, Culture and Subsistence of Humans in the Caucasus Between 40,000 and 10,000 Years Ago*. (Cambridge Scholars Publisher, 2020).

4. Golovanova, L. V. *et al.* Significance of ecological factors in the middle to upper paleolithic transition. *Curr. Anthropol.* **51**, 655–691 (2010).

5. Skinner, A. R. *et al.* ESR dating at Mezmaiskaya Cave, Russia. *Appl. Radiat. Isot.* **62**, 219–224 (2005).

6. Pinhasi, R., Higham, T. F. G., Golovanova, L. V. & Doronichev, V. B. Revised age of late Neanderthal occupation and the end of the Middle Paleolithic in the northern Caucasus. *Proc. Natl. Acad. Sci. U. S. A.* **108**, 8611–8616 (2011).

7. Hajdinjak, M. *et al.* Reconstructing the genetic history of late Neanderthals. *Nature* **555**, 652–656 (2018).

8. Golovanova, L. V., Hoffecker, J. F., Kharitonov, V. M. & Romanova, G. P. Mezmaiskaya cave: A neanderthal occupation in the northern caucasus. *Curr. Anthropol.* **40**, 77–86 (1999).

9. Golovanova, L. V. & Doronichev, V. B. The Middle Paleolithic of the Caucasus. *J. World Prehistory* **17**, 71–140 (2003).

10. Cleghorn, B. M., Christie, W. H. & Dong, C. C. S. Root and Root Canal Morphology of the Human Permanent Maxillary First Molar: A Literature Review. *Journal of Endodontics* **32**, 813–821 (2006).

11. Smith, T. M. *et al.* Dental evidence for ontogenetic differences between modern humans and Neanderthals. *Proc. Natl. Acad. Sci. U. S. A.* **107**, 20923–20928 (2010).

12. Becam, G. & Chevalier, T. Neandertal features of the deciduous and permanent teeth from Portel-Ouest Cave (Ariège, France). *Am. J. Phys. Anthropol.* **168**, 45–69 (2018).

13. Harvati, K. & Tourloukis, V. Human evolution in the southern Balkans. *Evol. Anthropol.* **22**, 43–45 (2013).

14. Gansauge, M.-T. *et al.* Single-stranded DNA library preparation from highly degraded DNA using T4 DNA ligase. *Nucleic Acids Res.* **45**, e79 (2017).

15. Schubert, M., Lindgreen, S. & Orlando, L. AdapterRemoval v2: Rapid adapter trimming, identification, and read merging. *BMC Res. Notes* **9**, 1–7 (2016).

16. Andrews, R. M. *et al.* Reanalysis and revision of the Cambridge reference sequence for human mitochondrial DNA. *Nat. Genet.* **23**, 147 (1999).

17. Green, R. E. *et al.* A Complete Neandertal Mitochondrial Genome Sequence Determined by High-Throughput Sequencing. *Cell* **134**, 416–426 (2008).

18. Li, H. & Durbin, R. Fast and accurate short read alignment with Burrows-Wheeler transform. *Bioinformatics* **25**, 1754–60 (2009).

19. Schubert, M. *et al.* Improving ancient DNA read mapping against modern reference genomes. *BMC Genomics* **13**, 178 (2012).

20. Picard Toolkit. Picard Toolkit. *Broad Institute, GitHub repository* (2019). Available at: http://broadinstitute.github.io/picard/.

21. Jónsson, H., Ginolhac, A., Schubert, M., Johnson, P. L. F. & Orlando, L. mapDamage2.0: fast approximate Bayesian estimates of ancient DNA damage parameters. *Bioinformatics* **29**, 1682–4 (2013).

22. Peyrégne, S. & Peter, B. M. AuthentiCT: a model of ancient DNA damage to estimate the proportion of present-day DNA contamination. *Genome Biol.* **21**, (2020).

23. Peyrégne, S. *et al.* Nuclear DNA from two early Neandertals reveals 80,000 years of genetic continuity in Europe. *Sci. Adv.* **5**, 5873 (2019).

24. Meyer, M. *et al.* Nuclear DNA sequences from the Middle Pleistocene Sima de los Huesos hominins. *Nature* **531**, 504–507 (2016).

25. Prüfer, K. *et al.* The complete genome sequence of a Neanderthal from the Altai Mountains. *Nature* **505**, 43–49 (2014).

26. Mafessoni, F. *et al.* A high-coverage neandertal genome from chagyrskaya cave. *Proc. Natl. Acad. Sci. U. S. A.* **117**, 15132–15136 (2020).

27. Meyer, M. *et al.* A high-coverage genome sequence from an archaic Denisovan individual. *Science (80-. ).* **338**, 222–226 (2012).

28. Prüfer, K. *et al.* A high-coverage Neandertal genome from Vindija Cave in Croatia. *Science (80-. ).* **358**, 655–658 (2017).

29. Paten, B. *et al.* Genome-wide nucleotide-level mammalian ancestor reconstruction. *Genome Res.* **18**, 1829–1843 (2008).

30. Paten, B., Herrero, J., Beal, K., Fitzgerald, S. & Birney, E. Enredo and Pecan: Genome-wide mammalian consistency-based multiple alignment with paralogs. *Genome Res.* **18**, 1814–1828 (2008).

31. Herrero, J. *et al.* Ensembl comparative genomics resources. *Database* **2016**, (2016).

32. Danecek, P. *et al.* Twelve years of SAMtools and BCFtools. *Gigascience* **10**, (2021).

33. Li, H. A statistical framework for SNP calling, mutation discovery, association mapping and population genetical parameter estimation from sequencing data. *Bioinformatics* **27**, 2987–2993 (2011).

34. Purcell, S. *et al.* PLINK: A tool set for whole-genome association and population-based linkage analyses. *Am. J. Hum. Genet.* **81**, 559–575 (2007).

35. Patterson, N. *et al.* Ancient admixture in human history. *Genetics* **192**, 1065–1093 (2012).

36. Renaud, G., Slon, V., Duggan, A. T. & Kelso, J. Schmutzi: Estimation of contamination and endogenous mitochondrial consensus calling for ancient DNA. *Genome Biol.* **16**, (2015).

37. Slon, V. *et al.* Neandertal and Denisovan DNA from Pleistocene sediments. *Science (80-. ).* **356**, 605–608 (2017).

38. Krause, J. *et al.* The complete mitochondrial DNA genome of an unknown hominin from southern Siberia. *Nature* **464**, 894–897 (2010).

39. Reich, D. *et al.* Genetic history of an archaic hominin group from Denisova cave in Siberia. *Nature* **468**, 1053–1060 (2010).

40. Sawyer, S. *et al.* Nuclear and mitochondrial DNA sequences from two Denisovan individuals. *Proc. Natl. Acad. Sci. U. S. A.* **112**, 15696–15700 (2015).

41. Meyer, M. *et al.* A mitochondrial genome sequence of a hominin from Sima de los Huesos. *Nature* **505**, 403–406 (2014).

42. Ingman, M., Kaessmann, H., Pääbo, S. & Gyllensten, U. Mitochondrial genome variation and the origin of modem humans. *Nature* **408**, 708–713 (2000).

43. Katoh, K. & Standley, D. M. MAFFT multiple sequence alignment software version 7: Improvements in performance and usability. *Mol. Biol. Evol.* **30**, 772–780 (2013).

44. Drummond, A. J., Suchard, M. A., Xie, D. & Rambaut, A. Bayesian phylogenetics with BEAUti and the BEAST 1.7. *Mol. Biol. Evol.* **29**, 1969–1973 (2012).

45. Picin, A. *et al.* New perspectives on Neanderthal dispersal and turnover from Stajnia Cave (Poland). *Sci. Rep.* **10**, (2020).

46. Romandini, M. *et al.* A late Neanderthal tooth from northeastern Italy. *J. Hum. Evol.* **147**, (2020).

47. Posada, D. jModelTest: Phylogenetic model averaging. *Mol. Biol. Evol.* **25**, 1253–1256 (2008).

48. Fu, Q. *et al.* A revised timescale for human evolution based on ancient mitochondrial genomes. *Curr. Biol.* **23**, 553–559 (2013).

49. Douka, K. *et al.* Age estimates for hominin fossils and the onset of the Upper Palaeolithic at Denisova Cave. *Nature* **565**, 640–644 (2019).

50. Arsuaga, J. L. *et al.* Neandertal roots: Cranial and chronological evidence from Sima de los Huesos. *Science (80-. ).* **344**, 1358–1363 (2014).

51. Rambaut, A. FigTree v. 1.4.4. *http://tree.bio.ed.ac.uk/software/figtree/* (2018).

52. Weissensteiner, H. *et al.* HaploGrep 2: mitochondrial haplogroup classification in the era of high-throughput sequencing. *Nucleic Acids Res.* **44**, W58-63 (2016).

53. Bokelmann, L. *et al.* A genetic analysis of the Gibraltar Neanderthals. *Proc. Natl. Acad. Sci. U. S. A.* **116**, 15610–15615 (2019).

54. Vernot, B. *et al.* Unearthing Neanderthal population history using nuclear and mitochondrial DNA from cave sediments. *Science.* **372**, (2021).

55. Zavala, E. I. *et al.* Pleistocene sediment DNA reveals hominin and faunal turnovers at Denisova Cave. *Nature* (2021). doi:10.1038/s41586-021-03675-0

56. Dalén, L. *et al.* Partial genetic turnover in neandertals: Continuity in the east and population replacement in the West. *Mol. Biol. Evol.* **29**, 1893–1897 (2012).

57. Krause, J. *et al.* Neanderthals in central Asia and Siberia. *Nature* **449**, 902–904 (2007).

58. McLaren, W. *et al.* The Ensembl Variant Effect Predictor. *Genome Biol. 2016 171* **17**, 1–14 (2016).

59. Xing, G. *et al.* Mitochondrial 12S rRNA A827G mutation is involved in the genetic susceptibility to aminoglycoside ototoxicity. *Biochem. Biophys. Res. Commun.* **346**, 1131–1135 (2006).

60. Chaig, M. R. *et al.* A mutation in mitochondrial 12S rRNA, A827G, in Argentinean family with hearing loss after aminoglycoside treatment. *Biochem. Biophys. Res. Commun.* **368**, 631–636 (2008).

61. Ying, Z. *et al.* Mitochondrial haplogroup B increases the risk for hearing loss among the Eastern Asian pedigrees carrying 12S rRNA 1555A>G mutation. *Protein and Cell* **6**, 844–848 (2015).

62. Elson, J. L. *et al.* The presence of highly disruptive 16S rRNA mutations in clinical samples indicates a wider role for mutations of the mitochondrial ribosome in human disease. *Mitochondrion* **25**, 17–27 (2015).

63. Smith, P. M. *et al.* The role of the mitochondrial ribosome in human disease: Searching for mutations in 12s mitochondrial rRNA with high disruptive potential. *Hum. Mol. Genet.* **23**, 949–967 (2014).

64. Sonney, S. *et al.* Predicting the pathogenicity of novel variants in mitochondrial tRNA with MitoTIP. *PLoS Comput. Biol.* **13**, e1005867 (2017).

65. Pereira, L. *et al.* The Diversity Present in 5140 Human Mitochondrial Genomes. *Am. J. Hum. Genet.* **84**, 628–640 (2009).

66. Bolze, A. *et al.* A catalog of homoplasmic and heteroplasmic mitochondrial DNA variants in humans. *bioRxiv* 798264 (2019). doi:10.1101/798264

67. Elson, J. L., Turnbull, D. M. & Howell, N. Comparative Genomics and the Evolution of Human Mitochondrial DNA: Assessing the Effects of Selection. *Am. J. Hum. Genet.* **74**, 229–238 (2004).

68. Mishmar, D. *et al.* Natural selection shaped regional mtDNA variation in humans. *Proc. Natl. Acad. Sci. U. S. A.* **100**, 171–176 (2003).

69. Ruiz-Pesini, E., Mishmar, D., Brandon, M., Procaccio, V. & Wallace, D. C. Effects of Purifying and Adaptive Selection on Regional Variation in Human mtDNA. *Science (80-. ).* **303**, 223–226 (2004).

70. Rocha, H. *et al.* About the ‘pathological’ role of the mtDNA T3308C mutation... [1]. *Am. J. Hum. Genet.* **65**, 1457–1459 (1999).

71. Bordes, F. Mousterian cultures in France. *Science (80-. ).* **134**, 803–810 (1961).

72. Monnier, G. F. & Missal, K. Another Mousterian Debate? Bordian facies, chaîne opératoire technocomplexes, and patterns of lithic variability in the western European Middle and Upper Pleistocene. *Quat. Int.* **350**, 59–83 (2014).

73. Kolobova, K. A. *et al.* Archaeological evidence for two separate dispersals of Neanderthals into southern Siberia. *Proc. Natl. Acad. Sci. U. S. A.* **117**, 2879–2885 (2020).

74. Weiss, M., Lauer, T., Wimmer, R. & Pop, C. M. The Variability of the Keilmesser-Concept: a Case Study from Central Germany. *J. Paleolit. Archaeol.* **1**, 202–246 (2018).

75. Kozłowski, J. K. Middle palaeolithic variability in Central Europe: Mousterian vs Micoquian. *Quat. Int.* **326**–**327**, 344–363 (2014).

76. Rybin, E. & Kolobova, K. The Middle Palaeolithic of Altai: Variability and Evolution (In Russian). *Strat. plus* **1**, 33–78

77. Robinson, P. & Zemo jtel, T. Integrative genomics viewer (IGV): Visualizing alignments and variants. in *Computational Exome and Genome Analysis* 233–245 (2018). doi:10.1201/9781315154770-17

78. Kumar, S., Stecher, G., Li, M., Knyaz, C. & Tamura, K. MEGA X: Molecular evolutionary genetics analysis across computing platforms. *Mol. Biol. Evol.* **35**, 1547–1549 (2018).

79. Rosenbloom, K. R. *et al.* The UCSC Genome Browser database: 2015 update. *Nucleic Acids Res.* **43**, D670–D681 (2015).

80. Kumar, S., Stecher, G., Li, M., Knyaz, C. & Tamura, K. MEGA X: Molecular Evolutionary Genetics Analysis across Computing Platforms | Molecular Biology and Evolution | Oxford Academic. *Mol. Biol. Evol.* **35**, 1547–1549 (2018).

81. Fu, Q. *et al.* DNA analysis of an early modern human from Tianyuan Cave, China. *Proc. Natl. Acad. Sci. U. S. A.* **110**, 2223–2227 (2013).

82. Krause, J. *et al.* A Complete mtDNA Genome of an Early Modern Human from Kostenki, Russia. *Curr. Biol.* **20**, 231–236 (2010).

83. Fewlass, H. *et al.* Direct radiocarbon dates of mid Upper Palaeolithic human remains from Dolní Věstonice II and Pavlov I, Czech Republic. *J. Archaeol. Sci. Reports* **27**, (2019).

84. Ermini, L. *et al.* Complete Mitochondrial Genome Sequence of the Tyrolean Iceman. *Curr. Biol.* **18**, 1687–1693 (2008).

85. Gilbert, M. T. P. *et al.* Paleo-Eskimo mtDNA genome reveals matrilineal discontinuity in Greenland. *Science (80-. ).* **320**, 1787–1789 (2008).

86. Fu, Q. *et al.* Genome sequence of a 45,000-year-old modern human from western Siberia. *Nature* **514**, 445–449 (2014).

87. Rougier, H. *et al.* Neandertal cannibalism and Neandertal bones used as tools in Northern Europe. *Sci. Rep.* **6**, (2016).

88. Briggs, A. W. *et al.* Targeted retrieval and analysis of five neandertal mtDNA genomes. *Science (80-. ).* **325**, 318–321 (2009).

89. Schmitz, R. W. *et al.* The Neandertal type site revisited: Interdisciplinary investigations of skeletal remains from the Neander Valley, Germany. *Proc. Natl. Acad. Sci. U. S. A.* **99**, 13342–13347 (2002).

90. Serre, D. *et al.* No evidence of Neandertal mtDNA contribution to early modern humans. *PLoS Biol.* **2**, (2004).

91. Semal, P. *et al.* New data on the late neandertals: Direct dating of the belgian spy fossils. *Am. J. Phys. Anthropol.* **138**, 421–428 (2009).
